# Neurophysiological alterations in mice and humans carrying mutations in *APP* and *PSEN1* genes

**DOI:** 10.1186/s13195-023-01287-6

**Published:** 2023-08-22

**Authors:** Fran C. van Heusden, Anne M. van Nifterick, Bryan C. Souza, Arthur S. C. França, Ilse M. Nauta, Cornelis J. Stam, Philip Scheltens, August B. Smit, Alida A. Gouw, Ronald E. van Kesteren

**Affiliations:** 1 Department of Molecular and Cellular Neurobiology, Center for Neurogenomics and Cognitive Research, Vrije Universiteit Amsterdam, Amsterdam, 1081HV The Netherlands; 2 Alzheimer Center Amsterdam, Neurology, Vrije Universiteit Amsterdam, Amsterdam UMC Location VUmc, Amsterdam, 1081HV The Netherlands; 3 Clinical Neurophysiology and MEG Center, Neurology, Vrije Universiteit Amsterdam, Amsterdam UMC Location VUmc, Amsterdam, 1081HV The Netherlands; 4 Donders Institute for Brain, Cognition and Behavior, Radboud University, Nijmegen, 6525AJ The Netherlands; 5 Netherlands Institute for Neuroscience, Royal Netherlands Academy of Arts and Sciences, Amsterdam, 1105 BA The Netherlands; 6 MS Center Amsterdam, Neurology, Vrije Universiteit Amsterdam, Amsterdam UMC Location VUmc, Amsterdam, 1081HV The Netherlands

**Keywords:** Translational network neuroscience, Autosomal dominant Alzheimer’s disease, Mouse models of Alzheimer’s disease, Local field potential (LFP) recordings, Functional brain imaging

## Abstract

**Background:**

Studies in animal models of Alzheimer’s disease (AD) have provided valuable insights into the molecular and cellular processes underlying neuronal network dysfunction. Whether and how AD-related neurophysiological alterations translate between mice and humans remains however uncertain.

**Methods:**

We characterized neurophysiological alterations in mice and humans carrying AD mutations in the *APP* and/or *PSEN1* genes, focusing on early pre-symptomatic changes. Longitudinal local field potential recordings were performed in APP/PS1 mice and cross-sectional magnetoencephalography recordings in human *APP* and/or *PSEN1* mutation carriers. All recordings were acquired in the left frontal cortex, parietal cortex, and hippocampus. Spectral power and functional connectivity were analyzed and compared with wildtype control mice and healthy age-matched human subjects.

**Results:**

APP/PS1 mice showed increased absolute power, especially at higher frequencies (beta and gamma) and predominantly between 3 and 6 moa. Relative power showed an overall shift from lower to higher frequencies over almost the entire recording period and across all three brain regions. Human mutation carriers, on the other hand, did not show changes in power except for an increase in relative theta power in the hippocampus. Mouse parietal cortex and hippocampal power spectra showed a characteristic peak at around 8 Hz which was not significantly altered in transgenic mice. Human power spectra showed a characteristic peak at around 9 Hz, the frequency of which was significantly reduced in mutation carriers. Significant alterations in functional connectivity were detected in theta, alpha, beta, and gamma frequency bands, but the exact frequency range and direction of change differed for APP/PS1 mice and human mutation carriers.

**Conclusions:**

Both mice and humans carrying *APP* and/or *PSEN1* mutations show abnormal neurophysiological activity, but several measures do not translate one-to-one between species. Alterations in absolute and relative power in mice should be interpreted with care and may be due to overexpression of amyloid in combination with the absence of tau pathology and cholinergic degeneration. Future studies should explore whether changes in brain activity in other AD mouse models, for instance, those also including tau pathology, provide better translation to the human AD continuum.

**Supplementary Information:**

The online version contains supplementary material available at 10.1186/s13195-023-01287-6.

## Background

Alzheimer’s disease (AD) is characterized by neuronal network alterations. In patients, oscillatory slowing and aberrant long-range functional connectivity are observed [1–4]. Similarly, mouse models of AD show changes in neuronal excitability and long-range connectivity [5–7]. Stimulating GABAergic neurotransmission could mitigate these changes [8]. In addition, alterations in gamma oscillatory activity have been observed in AD [9] and in mouse models of AD [10] and could be reversed by restoring sodium channel expression in parvalbumin (PV)-positive GABAergic interneurons or optogenetic entrainment of PV interneuron firing in mice [10, 11].

Based on these findings, treatments that aim at restoring neuronal network function are currently being investigated. Restoring PV interneuron activity using neuronal transplants [12] or sensory stimulation [13, 14] reduces amyloid load, enhances gamma oscillatory activity and improves cognition in mice. Pharmacological approaches aimed at restoring excitation/inhibition balance, such as anti-epileptic drugs, are also being investigated [15, 16]. Furthermore, deep brain stimulation (DBS), transcranial magnetic stimulation (TMS), and transcranial current stimulation (TCS) all have yielded promising results both in mouse models of AD as well as in AD patients [17, 18].

Even though brain stimulation approaches provide exciting opportunities for AD treatment, knowledge on how neuronal network alterations in mice translate to the human AD continuum is still limited. As treatments have been suggested to be most effective during early disease stages [19], translation of findings at preclinical stages in particular is essential to guide the development of effective treatment protocols. However, comparison of mouse and human data is currently challenging as the majority of studies investigating neuronal network activity in AD mouse models have been performed at a single time point or over a limited period of time or have focused on a single frequency band. In addition, limited data is available on network alterations in humans during pre-symptomatic AD stages. In particular, this knowledge gap pertains to alterations in the hippocampus, which in humans is less accessible using EEG. Addressing this knowledge gap, we performed longitudinal local field potential (LFP) recordings in a commonly used mouse model of AD, APPswe/PSEN1dE9 (APP/PS1) mice [20], and collected magnetoencephalography (MEG) measurements from pre-symptomatic human subjects carrying *APP* or *PSEN1* mutations causing autosomal dominant familial AD. We used APP/PS1 double-transgenic mice because mutant *PSEN1* single-transgenic mice do not display amyloid pathology [21] and therefore are not a good model for human *PSEN1* mutation carriers. Both LFP and MEG measure the consequences of synchronized activity of pyramidal neurons [22], and combining LFP in mice and EEG/MEG in humans is a valuable approach for investigating neuronal mechanisms and understanding brain function [23–25]. LFP and MEG are direct measures of brain function, which allow for an objective and accurate identification of abnormal brain function preceding cognitive impairment in AD. By measuring oscillatory activity and functional connectivity across a wide range of frequencies longitudinally in mice and pre-symptomatically in humans, we aim to provide insight into whether and how AD-related neurophysiological alterations translate between mice and humans. We hypothesize that somewhere along the disease trajectory, mouse models of AD recapitulate neurophysiological changes that reflect early disease stages in humans.

This study is the first to report oscillatory activity and functional connectivity changes in an AD mouse model longitudinally over an extended time course, and the first to report MEG measurements in pre-symptomatic AD mutation carriers. Longitudinal LFP recordings started when mice were 3 months old and were performed weekly over a period of 9 months. APP/PS1 mice start developing amyloid plaques between 5 and 6 months of age [20], and the onset of cognitive impairment is usually reported between 6 and 12 months of age [26], suggesting that the first three recording months represent a pre-symptomatic stage, although occasionally memory deficits are reported as early as 4 months of age [27]. Cross-sectional MEG recordings were obtained of subjects carrying *APP* or *PSEN1* mutations who had no signs of cognitive decline yet. Multiple parameters of neurophysiological activity were analyzed, including absolute and relative power, peak frequency, and long-range connectivity. Importantly, all parameters were computed for the same brain regions in both species, i.e., frontal cortex, parietal cortex, and hippocampus. These brain regions have previously been shown to exhibit altered activity in AD mouse models [28–34] and humans with AD [3, 4, 35–38]. By performing an extensive comparative analysis of mouse and human data we aim to understand whether and how AD-related oscillatory activity and functional connectivity alterations translate between mice and humans and which alterations may provide robust markers for the earliest stages of AD.

## Methods

### Animals

Male APP/PS1-PV-Cre mice (a cross of APP/PS1 and PV-Cre mice) were used. APP/PS1 mice [The Jackson Laboratory; strain B6C3-Tg(APPswe,PSEN1dE9)85Dbo/J with stock number 004462] are double-transgenic mice that harbor a chimeric human/mouse *APP* gene (Mo/HuAPP695swe) as well as a mutant human *PSEN1* gene with a deletion of exon 9 (PS1dE9) [39]. Both transgenes are controlled by the mouse prion protein promoter. PV-Cre mice [The Jackson Laboratory; Strain B6.129P2-Pvalbtm1(cre)Arbr/J with stock number 017320] express Cre recombinase under the endogenous parvalbumin promoter. Mouse lines were maintained on a C57BL/6JCrl background (Charles River Laboratories). APP/PS1-PV-Cre mice were used to allow for future PV interneuron-specific interventions. Previous studies showed that these mice are indistinguishable from APP/PS1 mice in the absence of Cre-dependent interventions [27]. Mice had ad libitum access to food and water and were kept on a 12-h light–dark cycle. All experiments were approved by the Central Committee for Animal Experiments and the Animal Welfare Body of the Vrije Universiteit Amsterdam in full compliance with the directive 2010/63/EU.

### LFP

#### Electrode arrays and surgery

Custom-made electrode arrays were assembled as previously described [40]. In short, tungsten (99.95%) CS SIS insulated wires (California Fine Wire, CFW2033234) with a diameter of 50.8 μm were aligned using custom-made alignment grids with holes spaced at 250 μm and fixed into place using VivadentTetric Evoflow dental cement (Hofmeester, #073877) and dental LED curing light. Two electrode arrays were constructed and connected to a printed circuit board (PCB), designed using the electronic design automation software Eagle (Autodesk), [40] at appropriate distance from one another. An insulated stainless-steel wire connected to a stainless-steel screw (Jeveka, #840000A20010002) was soldered to the PCB to provide a ground. An Omnetics connector (MSA Components, #A79026-001) that had been soldered to the PCB allowed for coupling to the recording equipment. After electrode arrays were fixed into place with photo-activated glue, individual electrodes were placed into the holes of the PCB, stripped from their wire coat using a surgical blade, and silver paint was applied to connect them to the PCB. Finally, the PCB was covered with epoxy adhesive (Liqui Moly, #6183) for protection. Mice were 10–11 weeks old at the time of surgery. One day before surgery carprofen (0.067 mg/ml, RIMADYL Cattle) was added to the drinking water and 30 min prior to surgery Temgesic (0.05 mg/kg, Invidior) was injected subcutaneously. Anesthesia was induced with isoflurane and the mouse was placed in a stereotactic frame (KOPF, model 942). Following shaving, the skin was disinfected with ethanol and betadine, and lidocaine (2% lidocaine-HCl, Fresenius Kabi, #20805) was injected subcutaneously at the incision site. The skull was cleaned using a cotton swab using hydrogen peroxide (15% solution, Sigma, #216763), and then scratched with a scalpel blade to facilitate binding of the adhesive. Two windows were drilled in the skull on top of the left hemisphere. A stainless-steel screw (Jeveka, #840000A20010002) was placed in the skull on top of the cerebellum as reference and ground, and two additional screws were fixed on the left and right parietal bones to serve as anchors for the dental cement. Electrode arrays were lowered into the brain, such that 9 electrodes of the first array were located in the prefrontal cortex (coordinates: AP0.5–1.75, ML0.5–1.0, and DV-2.3 for the 3 anterior electrodes and DV1.9 for the 6 posterior electrodes; Supplementary Fig. 1). From the second array, 7 electrodes were targeted at the parietal cortex (coordinates: AP-2.0, ML0.5–2.0, DV-0.7) and 14 at the hippocampus (coordinates: AP-2.25–2.5, ML0.5–2.0, and DV-1.5). Vaseline was applied to the windows and electrodes were fixed to the skull with a layer of Sun Medical Superbond C&B Kit (Hofmeester, #075794), followed by a layer of acrylic cement (Simplex Rapid, Kemdent). Approximately 15 min prior to the end of the surgery, saline was injected subcutaneously to facilitate recovery. In addition, the mouse’s homecage was placed on a heating pad for approximately 30 min post-surgery. Carprofen (0.067 mg/ml, RIMADYL Cattle) was present in the drinking water for at least 2 days post-surgery.

#### LFP recording

LFP recordings started when mice were 3 months of age (moa) and continued until they were 12 moa. Experiments started with 16 transgenic mice and 14 wildtype littermates. At the end of the 9-month recording period, 9 APP/PS1-PV-Cre and 9 wildtype mice could still be used for recordings. Mice were allowed to recover for at least 10 days prior the start of LFP recordings. Homecage recordings of 10–15 min were acquired using an Open Ephys recording system [41]. The Omnetics connector was attached to an RHD 32-channel recording headstage (Intan Technologies, #C3324) which was connected to an acquisition board (Open Ephys) with an RHD ultra-thin SPI cable (Intan Technologies, #C3216). Electrophysiological data were acquired with a sampling frequency of 30 kHz. Mice were recorded weekly during the light phase, 2–6 h after the lights were switched on. Video recordings were made using an overhead camera (Logitech BRIO, #960–001106) at 30 fps. Videos were synchronized with LFP recordings using TTL pulses in MATLAB that were directly sent to Open Ephys.

#### Preprocessing

Independent component analysis (ICA) was used to identify potential sources of noise in LFP traces. Noise components were then excluded from the traces prior to LFP reconstruction (EEGLAB) [42]. To remove large noise deflections that were present in a small number of recordings and could not be excluded using ICA, LFP signals exceeding 15*median absolute deviation from the median were excluded, including 2 s at the start and end of the noise period.

#### Channel selection

For the prefrontal cortex the 5 medial electrodes were selected (Supplementary Fig. 1). For the parietal cortex 4 electrodes were selected (Supplementary Fig. 1). If one of these electrodes did not produce a clean signal, and the number of included electrodes per brain region dropped below 4, an adjacent electrode was selected. For time–frequency analysis, hippocampal electrodes were grouped based on their location relative to the pyramidal layer: supra-pyramidal, pyramidal, or infra-pyramidal. The location of an electrode was determined based on ripple amplitude and theta phase (Supplementary Fig. 2; see Supplementary Methods for details). Only pyramidal and supra-pyramidal electrodes were used for analysis.

#### Behavioral states

Videos were analyzed in Bonsai [43] to obtain the *x* and *y* coordinates of the animals. Location was determined for each video frame. Video frame rate was 30 Hz. In case the animal could not be tracked, NaN values were entered. Files containing the coordinates were loaded into MATLAB (version 9.6.0 (R2019a), Natick, Massachusetts: The MathWorks Inc.). Velocity of the animal was determined using Bonsai coordinates. In case of NaNs, *x* and *y* coordinates were interpolated if the NaN period spanned less than 1 s. Next, delta (1–5 Hz) and theta (5–10 Hz) were filtered (*eegfilt* function in EEGLAB) from a hippocampal channel (Supplementary Fig. 3). Delta and theta envelopes were calculated by taking the absolute values of a Hilbert transform. The mean delta and theta envelope and the delta/theta ratio were then determined for 5-s epochs. These values were *z*-scored based on the epochs in which the animals were moving (velocity > 1cm/s). Subsequently, behavioral states were assigned to each 5-s epoch based on delta/theta ratio and the velocity of the animal (Supplementary Fig. 3). The behavioral state was considered “awake moving” when velocity > 1cm/s. For subsequent analysis, only “awake moving” epochs with an average speed < 4 cm/s were included. Behavioral state was classified as “quiet wake” when velocity < 1 cm/s and the z-scored theta/delta ratio < 5, and as “sleep” when velocity < 1 cm/s and the z-scored theta/delta ratio > 5. If only one 5-s epoch was classified as sleep, the epoch was assigned as “quiet wake.”

### Human participants

Eleven subjects > 18 years of age with mutations in either *PSEN1* (*n* = 9) or *APP* (*n* = 2) (Supplementary Table 1) were recruited from the Amsterdam Dementia Cohort (ADC) of the Amsterdam UMC (location VUmc) [44], the Dutch DIAN study cohort, through familial AD patient communities and through word-of-mouth and internet advertisement. The estimated years before symptom onset (EYBSO) was defined as the difference between a participant’s age and the reported parental (or sibling) age of symptom onset. Cognitive performance was assessed by the Mini-Mental State Examination (MMSE) [45] and extensive neuropsychological testing (see Supplementary Methods for details). Psychiatric symptoms, subjective cognitive decline, and instrumental activities of daily living were evaluated (see Supplementary Methods for details). For each mutation carrier three sex- and age-matched healthy control subjects (total *n* = 33) with available 5 min eyes-closed resting-state MEG and brain MRI were retrospectively selected from other studies of the Amsterdam UMC (MANTA (2018.070), EMIF-AD (2014.2010), the MuMo Brain project (2018.330) and the Amsterdam MS Cohort [46]). Whenever possible, healthy subjects were matched on MEG scanner type, educational level, amyloid beta status (verified using amyloid PET or CSF examination), and Mini-Mental State Examination (MMSE). Control subjects with cognitive, neurological, or psychiatric disorders and the use of psychoactive medication at the time of measurement were excluded from analyses if data was available. All participants provided written informed consent for the use of their data for research purposes.

### MEG

#### Recording

Ten minutes of eyes-closed resting-state MEG was recorded in a magnetically shielded room using a 306-channel whole-head system (Elekta Neuromag Oy, Helsinki, Finland) at a sample frequency of 1250 Hz, an online anti-aliasing filter of 410 Hz, and high-pass filter of 0.1 Hz. Five head-position indicator coils and the outline of the participant’s scalp (± 500 points) were digitized using a 3D digitizer (Fastrak, Polhemus, Colchester, VT, USA) to determine the head position relative to the MEG sensors. MEG data was co-registered to the individual T1-weighted structural MRI scan. MEG recording was performed before or at least one week after MRI scanning to avoid potential magnetization artifacts. In April 2021, the system was replaced by a Triux Neo system (MEGIN Oy, Finland) with identical channel number and type, allowing combined use of data. Data for 3 mutation carriers and 4 healthy controls was acquired using the Triux Neo system at a sampling frequency of 1000 Hz, an online anti-aliasing filter of 330 Hz, and high-pass filter of 0.1 Hz. The scalp outline was obtained in a line-like manner (± 2500 points). Seven healthy control subjects were scanned on both systems for comparison (Supplementary Fig. 4). All subjects were in supine position during MEG recording and instructed to close their eyes, lie still, relax, and think of nothing in particular while staying alert.

#### Preprocessing

Sensor-space MEG data was preprocessed to obtain artifact-free source-level data of 90 regions (78 cortical and 12 subcortical) of the Automatic Anatomical Labelling (AAL)-atlas [47]. The digitized scalp outline was co-registered with the subject’s structural MRI using anatomical landmarks and the digitized head shape points. The sphere that best fitted the individual scalp surface was used as a volume conductor model. Channels with excessive artifacts (e.g., flat lines, squid-jumps), as well as the first second and last 10 s of the time series, were excluded for estimation of the temporal extension of Signal Space Separation coefficients (implemented in MaxFilter software, Elekta Neuromag Oy, version 2.2.15; Taulu and Simola, 2004/2005/2006) used to suppress environmental noise. Following broad band filtering (0.5–100 Hz), an atlas-based beamforming approach was applied as previously described [48] in order to obtain source-level MEG time series (see Supplementary Methods for details). Preprocessed data was downsampled and segmented in epochs of ± 13.13 s (Electa data) or ± 12.30 s (Triux Neo data), each with a length of 4096 samples. Ten epochs were visually selected by AN and/or dr. E.M.M. Strijbis (physician) [49] based on the absence of artifacts and drowsiness of the patient and used for further analyses. Data analysis was restricted to a total of 11 frontal, 5 parietal, and 1 hippocampal virtual electrode, all of the left hemisphere, to match the mouse LFP data (Supplementary Table 2).

### LFP and MEG analysis

#### Time–frequency analysis

Time–frequency decomposition of LFP and MEG data was performed using Morlet wavelets. Wavelets of which the peak frequency spanned from 1 to 120 Hz in 140 linear steps and showed a 3-Hz full-width at half maximum in the spectral domain were used to convolve the signal. In the temporal spectrum, a sliding window of 6 s was used. To plot the power spectral density (PSD), the time–frequency spectrum was averaged over the selected channels per brain region. Normalized power was calculated by dividing the power at each frequency by the total power over 1–120 Hz. LFP spectra were further averaged over behavior states (quiet wake or awake moving) and MEG spectra were averaged across epochs. Frequency band definitions and their functional or behavioral correlates of specific oscillations differ slightly between mice and humans. To be able to compare our data to previously published research, oscillations were defined using species-specific frequency bands. For mice, LFP was reported using frequency bands: delta (1–5 Hz), theta (5–10 Hz), alpha (10–13 Hz), beta (13–30 Hz), low gamma (30–60 Hz) and high gamma (60–120 Hz). Human MEG was reported using the frequency bands: delta (1–4 Hz), theta (4–8 Hz), alpha (8–13 Hz), beta (13–30 Hz), low gamma (30–60 Hz), and high gamma (60–100 Hz). Power-over-time plots were smoothed using a 3-point span (MATLAB *smooth* function). For absolute power, Triux Neo MEG data were excluded as that scanner produced different absolute power values (Supplementary Fig. 4).

#### Peak frequency

Time–frequency decomposition was performed as above, with Morlet wavelets of which the peak frequency spanned from 6 to 10 Hz (LFP) or 5 to 13 Hz (MEG) in steps of 0.1 Hz. Peak frequency was identified using the MATLAB *findpeaks* function. In case more than one peak was identified within the selected frequency range, the peak with the maximum amplitude was selected. For LFP data, to visualize peak frequency over time, theta peak frequency was first identified from individual mice and afterwards averaged per genotype. Plots were smoothed using a 3-point span.

#### Weighted phase lag index

The weighted phase lag index (wPLI) was used to compute functional connectivity between brain regions [50]. For each frequency, channels of each area were combined through a generalized eigendecomposition that maximized the covariance matrix of the narrow-band signal in comparison to the broad band [51]. The instantaneous phase of the corresponding weighted signal of each area was then computed using the Hilbert transform, and subsequently used to compute the wPLI [52]. A 1–120 Hz wPLI spectrum was constructed in 140 linear steps for frontal-parietal and frontal-hippocampal connections. Hippocampal-parietal connections were not analyzed due to close proximity of these brain regions in mice. Mean connectivity was calculated per frequency band, based on the combination of prior PSD bands and visually identified peak connectivity in the wPLI spectrum. Frequency bands for LFP data: delta (1–5 Hz), theta/alpha (5–13 Hz), beta 1 (13–20 Hz), beta 2 (20–30 Hz), low gamma (30–50 Hz), for MEG data: delta (1–4 Hz), theta/alpha (4–13 Hz), beta 1 (13–20 Hz), beta 2 (20–30 Hz), low gamma (30–50 Hz). Graphs were smoothed with a 3-point span.

#### Amplitude envelope correlation

Amplitude-based functional connectivity was computed using corrected amplitude envelope correlation (AECc) [53, 54]. In steps of 1 Hz, 1–50 Hz data were filtered using the EEGLAB *eegfilt* function, a Hilbert transform was applied and the amplitude envelope was extracted. Pair-wise Pearson correlations were then computed between the envelopes of each pair of time series. A pairwise leakage correction of the amplitude envelopes was applied using regression analysis. This orthogonalization was performed separately for each pair of time series in two directions, meaning time series X was regressed out from time series Y and time series Y was regressed out from time series X. The correlation between orthogonalized envelopes was averaged over both directions to obtain the AECc. AECc values were normalized to the range [0–1]. AECc was computed for all time series combinations and afterwards averaged per connection. AECc-over-time plots (LFP data only) were smoothed with a 3-point span. Frequency ranges were the same as for the wPLI analyses.

### Statistics

Longitudinal LFP data was analyzed by fitting a mixed model with Geisser-Greenhouse correction as implemented in Graphpad Prism (GraphPad Prism version 9.3.1, GraphPad Software, San Diego, CA, USA). Mixed-effects analysis assessed the effect of the fixed factors time, genotype, and time × genotype. All statistics are reported in supplementary tables. Significant main effects of genotype and time × genotype interaction effects are reported in figure legends and main text.

Due to the high number of time points and frequencies analyzed, post hoc comparisons (Šidák’s multiple comparisons test) after mixed-effects analysis were rarely significant. We therefore report uncorrected statistical test results (performed in MATLAB) in all figures. Normality of data was tested for using the Kolmogorov–Smirnov test and two-sample *t*-tests or Wilcoxon rank sum tests were performed accordingly. In cases of normal distribution but unequal variance, as determined by a two-sample *F*-test for equal variances, unequal variances *t*-tests were performed. The significance threshold was set at 0.05. In figures, significant results are indicated by red bars on the *x*-axis. In the main text, we only report results from uncorrected two-sample tests when a significant main or interaction effect was found in the mixed-effects analysis (see previous paragraph), unless explicitly mentioned otherwise.

Human demographic data was analyzed by non-parametric Mann–Whitney *U* tests or *X*^2^ tests when appropriate, using IBM SPSS statistics 26 (Armonk, NY: IBM Corp). MEG data was analyzed in MATLAB by two-sample t-tests or Wilcoxon rank sum tests depending on normal distribution of the data, tested with similar methods as described above. In cases of normal distribution but unequal variance, unequal variances *t*-tests were performed. The significance threshold was set at 0.05. Significant results are indicated by red bars and asterisks in figures, and test results are reported in the figure legends.

## Results

### Subjects

#### Mice

LFP recordings were performed in male APP/PS1 and wildtype control mice at 3–12 moa. Weekly LFP recordings were performed in the animal’s homecage. As awake mobility was the predominant behavioral state (Supplementary Fig. 5, Supplementary Table 3), the main text and figures will present the analysis of oscillatory changes during awake mobility. No behavioral signs of epileptic activity were observed in this state, which is in line with previous studies showing that epileptic discharges in APP/PS1 mice occur primarily during sleep [55].

#### Human participants

MEG recordings were obtained from 11 pre-symptomatic *APP* or *PSEN1* mutation carriers and 33 healthy controls. Table 1 presents group characteristics. Although multiple participants were close to or past their estimated age of symptom onset (EYBSO), none of the mutation carriers had an abnormal performance on neuropsychological examination (Supplementary Table 4).

**Table 1:** Demographics and neuropsychological data for human subjects

	**Mutation carriers**	**Healthy controls**	***P* value**
*N*	11	33	-
Age (years)	49 [20–61]	49 [20–62]	0.957
Female/male (*n*)	8/3	24/9	> 0.999
*PSEN1*/*APP* (*n*)	9/2	-	-
Education (Verhage)	6 [5–7]	6 [1–7]	0.742
Global cognition (MMSE)	29 [27–30] (*n* = 11)	27 [27–30] (*n* = 7)	0.375
Estimated years before symptom onset	1 [− 16–22]	-	-

Group median and range are presented unless otherwise specified. Education is presented in Verhage score (range 1–7); *MMSE*, Mini-Mental State Examination (max 30); negative values for estimated years before symptom onset indicate that subjects had passed the estimated age of symptom onset

### Absolute power

#### APP/PS1 mice show an age-dependent increase in absolute power

**Fig. 1:**
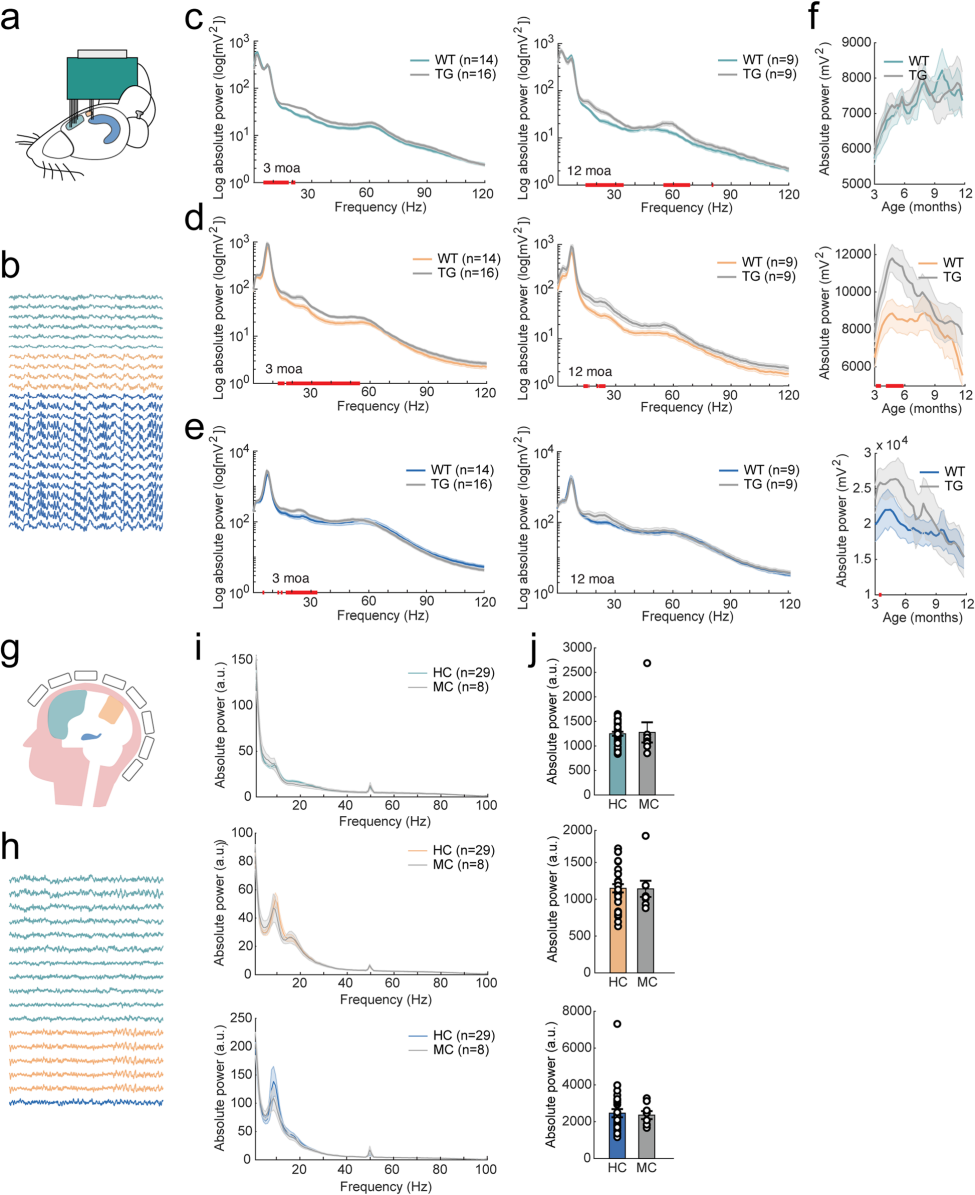
AD-related changes in total absolute power in APP/PS1 mice and human mutation carriers. For figure legends, ^ refers to a significant main effect of genotype; * refers to a significant interaction effect of genotype × time. Significant results from uncorrected two-sample tests are depicted by red bars on the *x*-axis. **a** LFP electrodes were located in the prefrontal cortex (green), parietal cortex (orange), and hippocampus (blue). A reference screw was placed at the cerebellum. **b** LFP example traces from the prefrontal cortex (green), parietal cortex (orange), and hippocampus (blue). **c**-**d** Mean PSD for 3-month-old (left) and 12-month-old (right) mice in the prefrontal cortex (**c**), parietal cortex (**d**), and hippocampus **(e)**. Note the prominent theta (5–10 Hz) peak in the parietal cortex and hippocampus. **f** Total absolute power (summed power over 1–120 Hz) in the prefrontal cortex (top), parietal cortex (middle), and hippocampus (bottom) in 3–12-month-old APP/PS1 (TG) and wildtype (WT) mice. Parietal cortex: *; hippocampus: *. **g** Graphical representation of MEG recordings in human participants. **h** Example traces of MEG recordings from the frontal cortex (green), parietal cortex (orange), and hippocampus (blue). **i** Mean PSD in the frontal cortex (top), parietal cortex (middle), and hippocampus (bottom) for mutation carriers (MC) and healthy controls (HC). Note the alpha (8–13 Hz) peak in the parietal cortex and hippocampus. **j** Total absolute power (summed power over 1–100 Hz) in the frontal cortex (top), parietal cortex (middle), and hippocampus (bottom) in *APP* and *PSEN1* mutation carriers. No changes in total absolute power were detected in the prefrontal cortex (*W* = 1.273, *p* = 0.203), parietal cortex (*W* = 0.350, *p* = 0.726), or hippocampus (*W* =  − 0.277, *p* = 0.782)

LFP was recorded from the prefrontal cortex, parietal cortex, and hippocampus of the left hemisphere (Fig. 1A, B, Supplementary Fig. 1). PSDs from the parietal cortex and hippocampus were characterized by a peak in the theta range, whereas PSDs from the prefrontal cortex showed maximum absolute power in the delta range (Fig. 1C–E; see Materials and Methods for definitions of mouse and human frequency bands). Total power did not differ between APP/PS1 and wildtype animals in the prefrontal cortex, but mixed-effects analysis showed a significant interaction of genotype × time in the parietal cortex and hippocampus (Fig. 1F; Supplementary Table 5). Uncorrected two-sample tests indicated an increase in parietal total power in transgenic mice at approximately 3–6 moa.

**Fig. 2:**
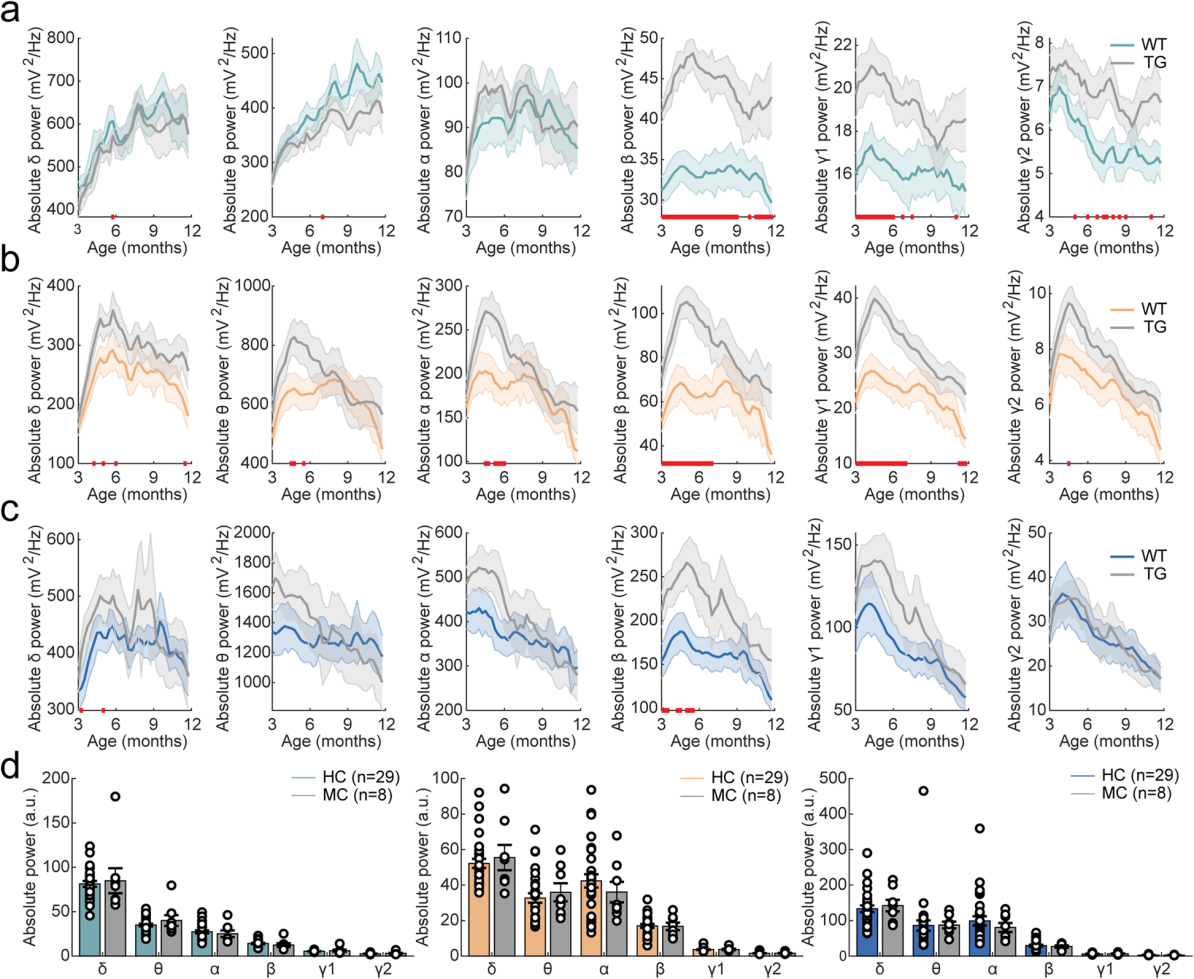
AD-related changes in absolute power per frequency band in APP/PS1 mice and human mutation carriers. In the legend, ^ refers to a significant main effect of genotype; * refers to a significant interaction effect of genotype × time. Significant results from uncorrected two-sample tests are depicted by red bars on the *x*-axis. **a**–**c** Mean absolute power per frequency band over the 9-month LFP recording period in the prefrontal cortex (*β*: ^*, *γ*1: ^) (**a**), in parietal cortex (*θ*: *, *α*: *, *β*: *, *γ*1: *) (**b**), and in hippocampus (*δ*: *, *θ*: *, *α*: *, *β*: *, *γ*1: *, *γ*2: *) (**c**). Frequency bands from left to right: delta (1–5 Hz), theta (5–10 Hz), alpha (10–13 Hz), beta (13–30 Hz), low gamma (30–60 Hz), and high gamma (60–120 Hz). **d** Quantification of absolute MEG power per frequency band in the frontal cortex (left), parietal cortex (middle), and hippocampus (right) in mutation carriers (MC) and healthy controls (HC). No changes in total absolute power were detected in the prefrontal cortex (*δ*: *W* = 0.867, *p* = 0.386; *θ*: *W* =  − 0.203, *p* = 0.839; *α*: *W* = 0.830, *p* = 0.406; *β*: *W* = 1.568, *p* = 0.117; *γ*1: *W* = 0.646, *p* = 0.519; *γ*2: *W* = 0.498, *p* = 0.618), parietal cortex (*δ*: *W* = 0.0184, *p* = 0.985; *θ*: *W* =  − 0.793, *p* = 0.428; *α*: *W* = 0.756, *p* = 0.449; β: *W* = 0.0922, *p* = 0.927; *γ*1: *W* =  − 0.461, *p* = 0.645; *γ*2: *W* = 0.0553, *p* = 0.956) or hippocampus (*δ*: *W* =  − 0.646, *p* = 0.519; *θ*: *W* =  − 1.310, *p* = 0.190; *α*: *W* = 0.535, *p* = 0.593; *β*: *W* = 0.0184, *p* = 0.985; *γ*1: *W* =  − 0.424, *p* = 0.671; *γ*2: *W* = 0.129, *p* = 0.897). Frequency bands from left to right: delta, (1–4 Hz), theta (4–8 Hz), alpha (8–13 Hz), beta (13–30 Hz), low gamma (30–60 Hz), and high gamma (60–100 Hz)

When calculating mean absolute power per frequency band, no pronounced differences were detected in delta, theta, or alpha power in the prefrontal cortex, but mixed-effects analysis showed that absolute power in the beta and low gamma range was increased in APP/PS1 animals (Fig. 2A, Supplementary Table 5). In the parietal cortex, theta, alpha, beta, and low gamma frequency bands showed a significant genotype × time interaction effect (Supplementary Table 5). Uncorrected two-sample tests indicated an increase in absolute power in these frequency bands at approximately 3–6 moa (Fig. 2B). In the hippocampus, all frequency bands showed a significant genotype × time interaction effect (Fig. 2C, Supplementary Table 5). Overall, APP/PS1 mice exhibited an increase in absolute power that was especially pronounced at early ages (3-6 moa) and at frequencies in the beta and low gamma range.

To exclude the possibility that genotypic differences in oscillatory power are caused by differences in velocity, mean velocity was calculated over the entire recording period (3-12 moa) (Supplementary Fig. 5). No main effect of genotype or interaction effect was found (Supplementary Fig. 5A). Moreover, spectral analyses performed during periods of quiet wake (Supplementary Fig. 6) revealed similar genotypic differences as observed during awake mobility, confirming that spectral changes are indeed independent of velocity or behavioral state.

#### Human *APP* and *PSEN1* mutation carriers have similar absolute power as age-matched controls

Source-reconstructed resting-state MEG data of left frontal cortical regions, parietal cortical regions, and the hippocampus were analyzed (Fig. 1G, H). Total absolute power was not different between mutation carriers and healthy controls (Fig. 1I, J). When absolute power was analyzed per frequency band, also no differences were found between mutation carriers and healthy controls (Fig. 2D). This differs from asymptomatic AD patients, which show a predominant increase in absolute power in delta and theta [56–61] and sometimes also in alpha frequencies [60, 62] across the brain, and decreased absolute beta power in posterior regions and the hippocampus [56, 59].

### Relative power

#### Relative power shifts from low to high frequencies in APP/PS1 mice

**Fig. 3:**
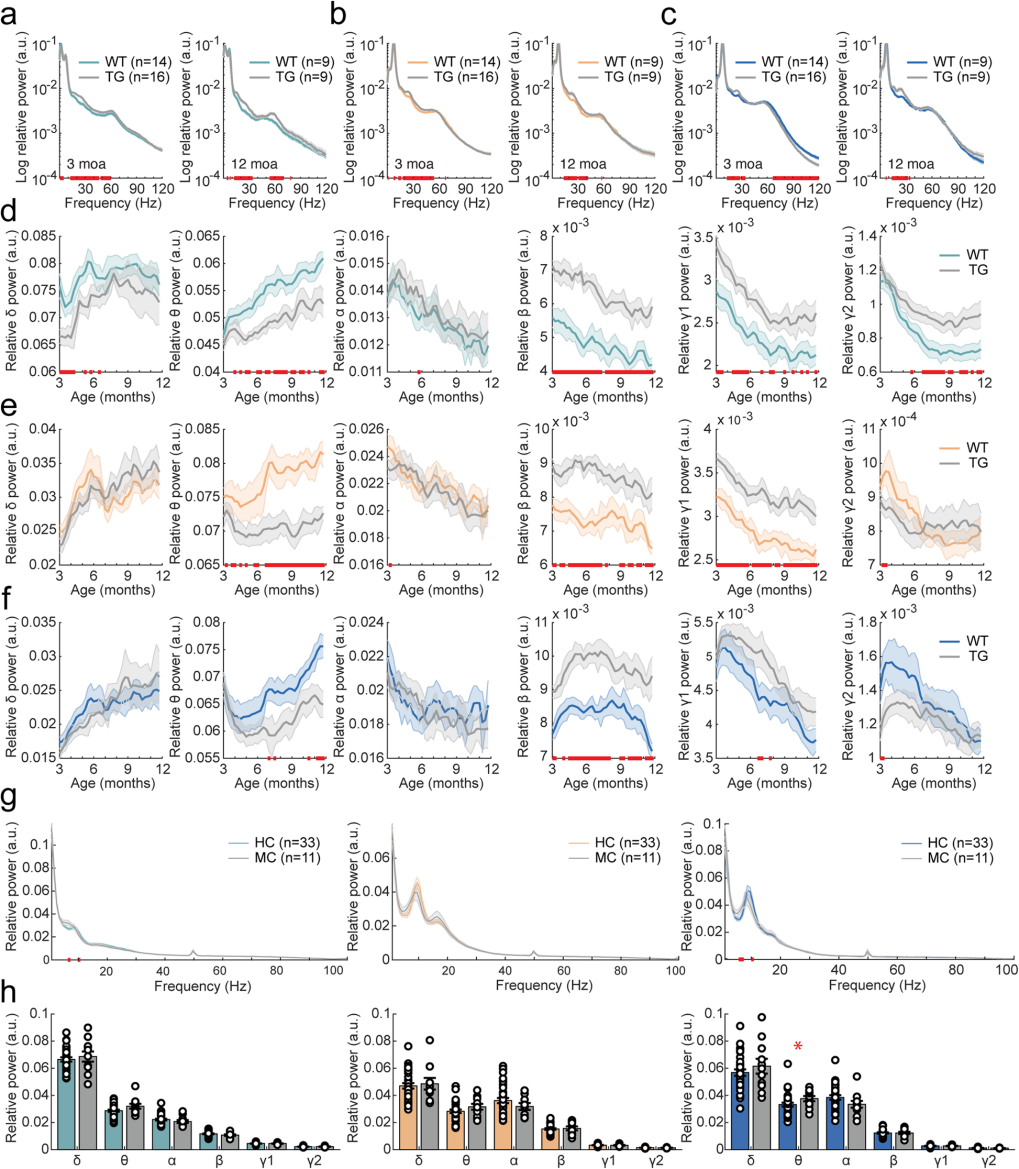
AD-related changes in relative power in APP/PS1 mice and human mutation carriers. In the legend, ^ refers to a significant main effect of genotype; * refers to a significant interaction effect of genotype × time. Significant results from uncorrected two-sample tests are depicted by red bars on the *x*-axis. **a**–**c** Mean PSD for 3-month-old (left) and 12-month-old (right) mice in the prefrontal cortex (**a**), parietal cortex (**b**), and hippocampus (**c**). **d**–**f** Mean relative power per frequency band over the 9-month LFP recording period in the prefrontal cortex (*θ*: ^; β: ^, *γ*1: ^) (**d**), in the parietal cortex (*θ*: ^; *β*: ^*, *γ*1: ^, *γ*2: *) (**e**), and in hippocampus (*δ*: *, *β*: ^, *γ*2: ^) (**f**). **g** Mean PSD in the frontal cortex (left), parietal cortex (middle), and hippocampus (right) for mutation carriers (MC) and healthy controls (HC). **h** Quantification of relative power in the frontal cortex (left), parietal cortex (middle), and hippocampus (right) of human mutation carriers per frequency band. No changes in relative power were detected in the frontal cortex (*δ*: *W* =  − 0.542, *p* = 0.588; *θ*: *W* =  − 1.680, *p* = 0.0929; *α*: *W* = 1.464, *p* = 0.143; *β*: *W* = 1.464, *p* = 0.143; *γ*1: *W* = 0.0271, *p* = 0.978; *γ*2: *W* =  − 0.0542, *p* = 0.957) or in the parietal cortex (*δ*: *W* =  − 0.190, *p* = 0.850; *θ*: *W* =  − 1.301, *p* = 0.193; *α*: *W* = 1.166, *p* = 0.244; *β*: *W* =  − 0.217, *p* = 0.828; *γ*1: *W* = 0.623, *p* = 0.533; *γ*2: *W* = 0.407, *p* = 0.684). In the hippocampus, power was increased in the theta frequency range, but not in other frequency ranges (*δ*: *W* =  − 0.596, *p* = 0.551; *θ*: *W* =  − 2.0599, *p* = 0.0394; *α*: *W* = 1.626, *p* = 0.104; *β*: *W* = 0.0271, *p* = 0.978; *γ*1: *W* = 0.379, *p* = 0.704; *γ*2: *W* = 0.949, *p* = 0.343)

Next, we analyzed relative power by dividing absolute power at each frequency by the total power (1–120 Hz) at the same timepoint. PSDs during awake mobility from the first and last recording, at 3 and 12 moa, respectively, showed a delta peak in the prefrontal cortex and theta peak in the parietal cortex and hippocampus (Fig. 3A–C). Additional peaks were observed in the beta and low gamma range. When analyzing relative power per frequency band, a shift of relative power from lower to higher frequencies was apparent. In the prefrontal cortex and parietal cortex, mixed-effects analysis showed that theta power was significantly reduced in APP/PS1 mice (Fig. 3D, Supplementary Table 6). In all brain regions, relative power was significantly increased in the beta frequency range, and in the prefrontal and parietal cortex also in the low gamma frequency range (Fig. 3D–F, Supplementary Table 6). Interestingly, despite the overall shift towards higher frequencies, high gamma relative power was reduced in the hippocampus of APP/PS1 mice and showed a significant genotype × time interaction effect in the parietal cortex. Uncorrected two-sample tests indicated an early reduction of high gamma relative power at 3 moa in these brain regions. Altogether, relative power seems to shift from low to high frequencies in APP/PS1 animals in a manner that is largely independent of age. A similar shift in relative power was observed during quiet wake (Supplementary Fig. 7).

#### Hippocampal relative theta power is increased in human *APP* and *PSEN1* mutation carriers

Relative power was analyzed for human MEG data over a frequency range of 1–100 Hz. PSDs showed a predominant peak in the alpha band in the parietal and hippocampal brain regions (Fig. 3G insets). When analyzing relative power per frequency band, a significant increase in relative theta power was observed in the hippocampus (Fig. 3G). Previous studies reported a similar increase in theta power in the hippocampus and cortical regions has been reported in symptomatic AD patients [35, 60, 63].

### Peak frequency

#### Theta peak frequency is largely unaltered in APP/PS1 mice

**Fig. 4:**
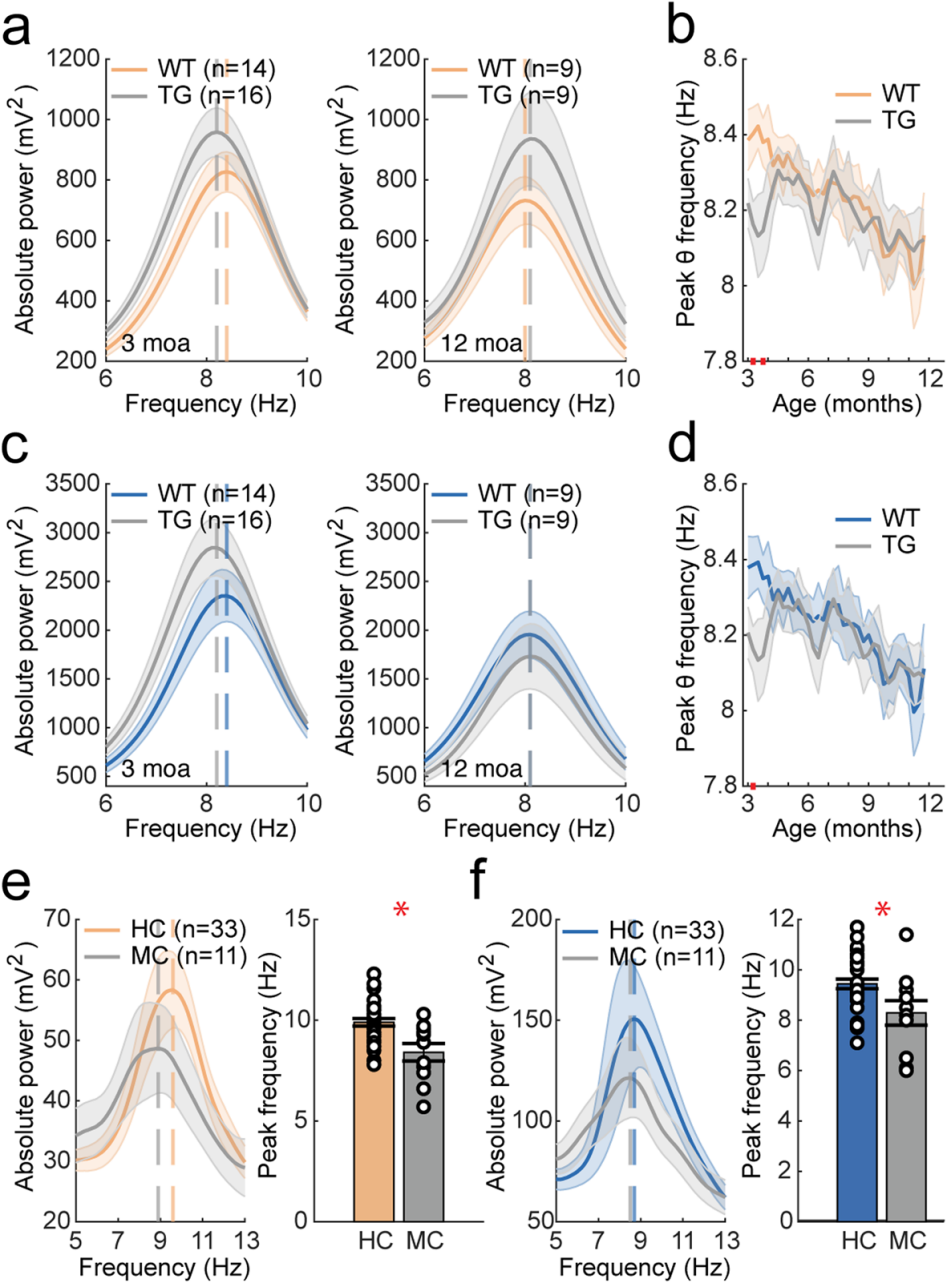
AD-related changes in peak frequency in awake moving APP/PS1 mice and human mutation carriers. In the legend, ^ refers to a significant main effect of genotype; * refers to a significant interaction effect of genotype × time. Significant results from uncorrected two-sample tests are depicted by red bars on the *x*-axis. **a** Mean PSD for theta frequencies over which theta (5–10 Hz) peak frequency was calculated in 3-month-old (left) and 12-month-old (right) mice in the parietal cortex. **b** Mean theta peak frequency over the 9-month recording period in the parietal cortex. Mixed-effects analysis did not show a main effect of genotype or a genotype × time interaction effect. **c** Mean PSD for theta frequencies in 3-month-old (left) and 12-month-old (right) mice in the hippocampus. **d** Mean theta peak frequency over the 9-month recording period in the hippocampus. Mixed-effects analysis did not show a main effect of genotype or a genotype × time interaction effect. **e** Left: mean PSD for the parietal cortex of human mutation carriers and healthy controls. Right: alpha (8–13 Hz) peak frequency is reduced in mutation carriers compared to healthy control subjects in the parietal cortex (*W* = 2.700, *p* = 0.00694). **f** Left: mean PSD for the hippocampus of human mutation carriers and healthy controls. Right: alpha peak frequency is reduced in mutation carriers compared to healthy control subjects in the hippocampus (*W* = 2.172, *p* = 0.0299)

As parietal and hippocampal PSDs in mice showed a characteristic theta peak, we subsequently analyzed theta peak frequency of APP/PS1 and wildtype animals in these brain regions (Fig. 4A, C). Mixed-effects analysis did not reveal any significant effects (Fig. 4B, D, Supplementary Table 7). Uncorrected two-sample tests however showed a transient decrease in peak frequency at 3 moa during awake mobility. During quiet wake, mixed-effects analysis did not show alterations in theta peak frequency (Supplementary Fig. 8).

#### Alpha peak frequency is reduced in human *APP* and *PSEN1* mutation carriers

Human MEG power spectra showed oscillatory peaks in the alpha frequency range in the parietal cortex and hippocampus (Fig. 4E, F). Mutation carriers had a significantly reduced alpha peak frequency in the parietal cortex and hippocampus compared to healthy controls, which is also observed in symptomatic AD patients [35, 60, 64].

### Functional connectivity

**Fig. 5:**
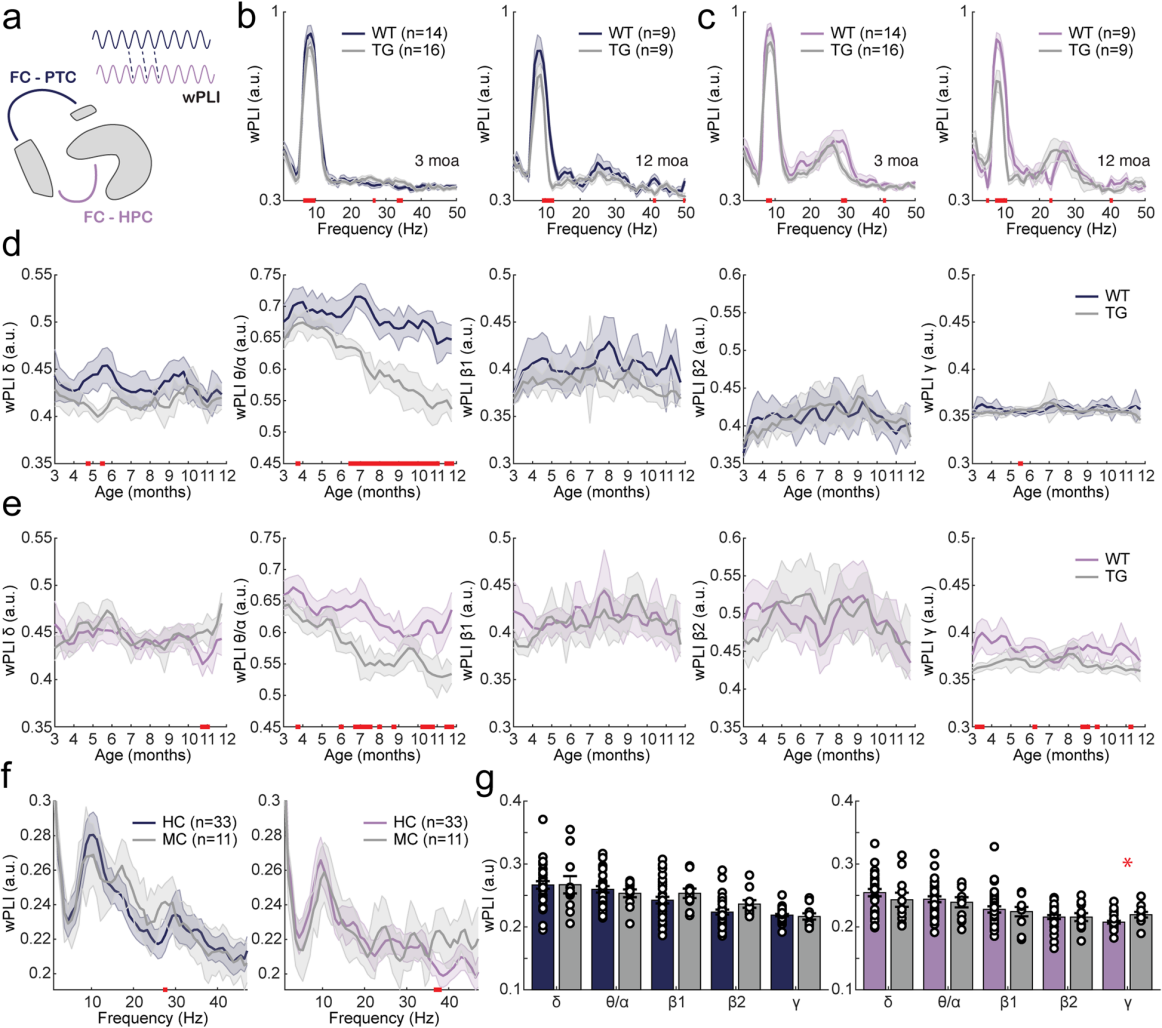
AD-related changes in phase-based connectivity, as measured by the weighted phase lag index (wPLI), in awake moving APP/PS1 mice and human mutation carriers. In the legend, ^ refers to a significant main effect of genotype; * refers to a significant interaction effect of genotype × time. Significant results from uncorrected two-sample tests are depicted by red bars on the *x*-axis. **a** Graphical representation of phase-based connectivity. Phase-based connectivity refers to the correlation of relative phase between two oscillatory signals. In the current study, the wPLI was analyzed for frontal cortex (FC)–parietal cortex (PTC) connections (dark blue) and for frontal cortex–hippocampus (HPC) connections (purple). **b**, **c** wPLI spectrogram for 3-month-old (left) and 12-month-old (right) mice for frontal cortex–parietal cortex (**b**) and frontal cortex–hippocampus (**c**). **d**, **e** Mean wPLI per frequency band over the 9-month recording period for frontal cortex–parietal cortex (*θ*/*α*: ^*) (**d**) and frontal cortex–hippocampus (*θ*/*α*: ^) (**e**). Frequency bands from left to right: delta (1–5 Hz), theta/alpha (5–13 Hz), beta 1 (13–20 Hz), beta 2 (20–30 Hz), gamma (30–50 Hz). **f** wPLI spectrograms for frontal cortex–parietal cortex (left) and frontal cortex–hippocampus (right) in human mutation carriers. **g** Quantification of phase-based connectivity between the frontal cortex and parietal cortex (left) and between the frontal cortex and hippocampus (right) per frequency band in mutation carriers (MC) and healthy controls (HC). Frequency bands within graphs from left to right: delta (1–4 Hz), theta/alpha (4–13 Hz), beta 1 (13–20 Hz), beta 2 (20–30 Hz), low gamma (30–50 Hz). No significant differences were found in wPLI between frontal cortex and parietal cortex (*δ*: *W* = 0.407, *p* = 0.684; *θ*/*α*: *W* = 0.352, *p* = 0.725; *β*1: *W* =  − 1.138, *p* = 0.255; *β*2: *W* =  − 1.870, *p* = 0.0615; *γ*: *W* = 1.138, *p* = 0.255) or frontal cortex and hippocampus (*δ*: *W* = 1.030, *p* = 0.303; *θ*/*α*: *W* = 0.271, *p* = 0.786; *β*1: *W* =  − 0.0542, *p* = 0.957; *β*2: *W* = 0.0813, *p* = 0.935), except for an increase in connectivity at gamma frequencies (*γ*: *W* =  − 2.467, *p* = 0.0136)

Long-range connectivity between brain regions was evaluated using the weighted phase lag index (wPLI) and corrected amplitude envelope correlation (AECc), which make use of oscillatory phase and amplitude, respectively. Connectivity was evaluated between (pre)frontal cortex and parietal cortex and between (pre)frontal cortex and hippocampus (Fig. 5A). wPLI and AECc were computed over a frequency range of 1–50 Hz, as it has previously been shown that connectivity computed at higher frequencies is unlikely to reflect biological processes [65].

#### APP/PS1 mice show an age-dependent decrease in phase- but not amplitude-based theta/alpha connectivity

During awake mobility, wPLI plotted over 1–50 Hz showed that the highest connectivity occurred within the theta and alpha frequency bands (5–13 Hz) (Fig. 5B, C). Two additional, but smaller peaks were observed in the beta 1 (13–20 Hz) and beta 2 (20–30 Hz) frequency range. When the spectrum was subdivided into frequency bands based on the location of peak connectivity, prefrontal-parietal connectivity (Fig. 5D) and prefrontal-hippocampal connectivity (Fig. 5E) showed similar patterns. Specifically, mixed-effects analysis showed that APP/PS1 animals exhibited a significant reduction in theta/alpha connectivity in both connections (Supplementary Table 8). In addition, a significant genotype × time interaction effect was found for theta/alpha connectivity between the prefrontal and parietal cortices. During quiet wake, a significant main effect of genotype for prefrontal-parietal theta-alpha connectivity was detected (Supplementary Fig. 9).

**Fig. 6:**
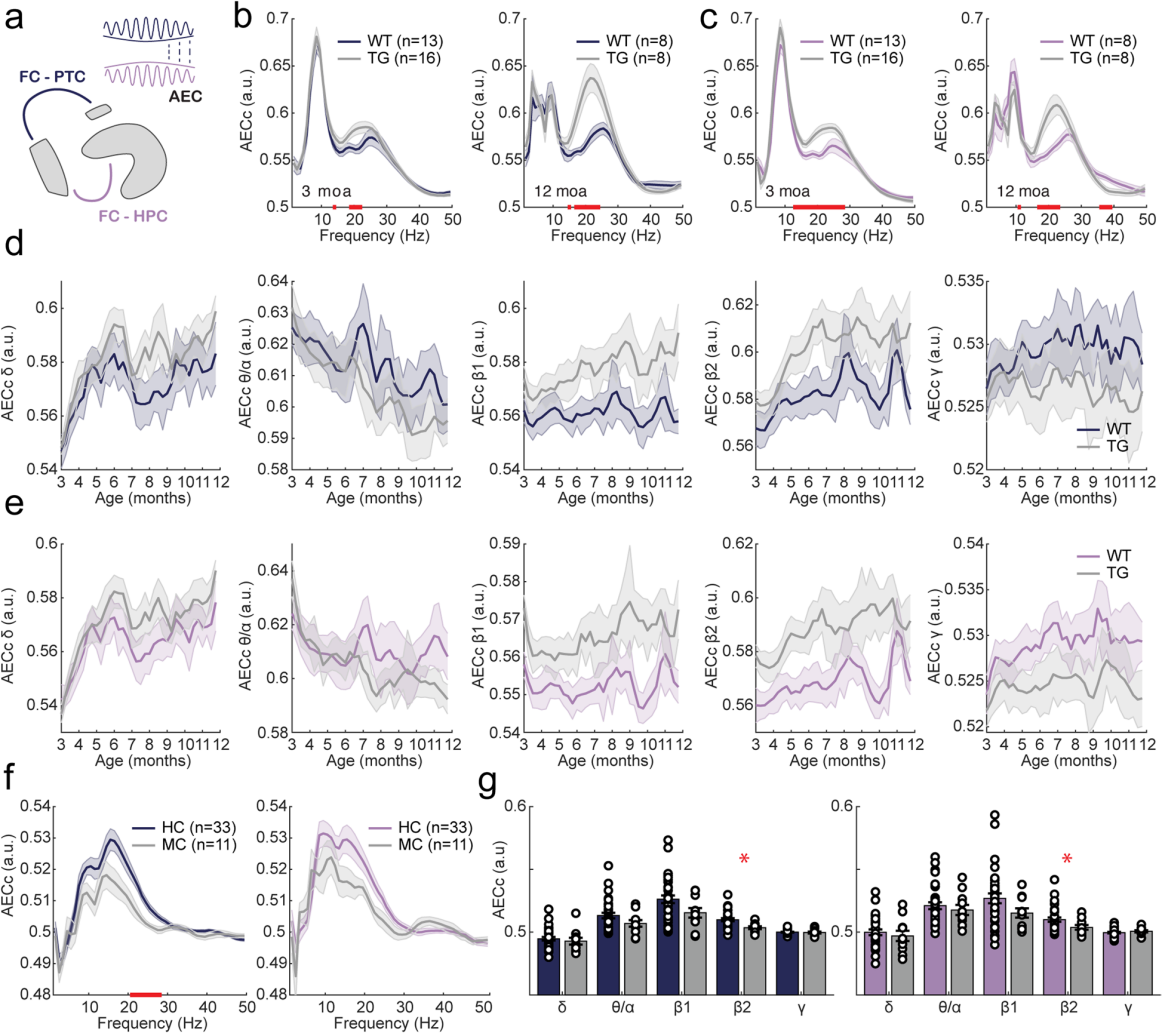
AD-related changes in amplitude-based connectivity, as measured by amplitude envelope correlation (AECc), in awake moving APP/PS1 mice and human mutation carriers. In the legend, ^ refers to a significant main effect of genotype; * refers to a significant interaction effect of genotype × time. Significant results from uncorrected two-sample tests are depicted by red bars on the *x*-axis. **a** Graphical representation of amplitude-based connectivity. Amplitude-based connectivity refers to the correlation of the amplitude of two oscillatory signals. In the current study, the AECc was analyzed for frontal cortex–parietal cortex connections (dark blue) and for frontal cortex–hippocampus connections (purple). **b** Frontal cortex–parietal cortex AECc spectrogram for 3-month-old (left) and 12-month-old (right) mice. **c** Frontal cortex–hippocampus AECc spectrogram for 3-month-old (left) and 12-month-old (right) mice. **d** Frontal cortex–parietal cortex mean AECc per frequency band over the 9-month recording period. *β*1: ^. **e** Frontal cortex–hippocampus mean AECc per frequency band over the 9-month recording period. *β*1: ^; *β*2: ^*. **f** Frontal cortex–parietal cortex (left) and frontal cortex–hippocampus (right) AECc spectrograms for human mutation carriers (MC) and controls (HC). **g** Quantification of amplitude-based connectivity between the frontal cortex and parietal cortex (left) and between the frontal cortex and hippocampus (right) per frequency band in humans. A decrease in AECc beta connectivity was found between the frontal cortex and parietal cortex, while other bands did not show significant differences (*δ*: W = 0.922, *p* = 0.357; *θ*/*α*: *W* = 1.247, *p* = 0.212; *β*1: *W* = 1.789, *p* = 0.0736; *β*2: *W* = 2.765, *p* = 0.00570; *γ*: *W* = 0.542, *p* = 0.588). Similarly, connectivity between the frontal cortex and hippocampus was decreased in the beta frequency band (*δ*: *W* = 0.515, *p* = 0.607; *θ*/*α*: *W* = 0.569, *p* = 0.569; *β*1: *W* = 1.708, *p* = 0.0877; *β*2: *W* = 2.0328, *p* = 0.0421; *γ*: *W* =  − 0.976, *p* = 0.329)

Interregional functional connectivity was also investigated using AECc (Fig. 6B, C). The metric was corrected for volume conduction to prevent spurious correlations. The spectrum was subdivided into the same frequency bands as for wPLI analysis. Mixed-effects analysis showed that connectivity was significantly increased in APP/PS1 mice in the beta 1 frequency band for both connections, and significant main and genotype × time effects were detected for prefrontal-hippocampal connectivity in the beta 2 frequency band (Fig. 6D, E; Supplementary Table 9). Similarly, during quiet wake, an increase in connectivity in the beta frequency band was detected between the prefrontal cortex and hippocampus (Supplementary Fig. 10).

#### Human *APP*/*PSEN1* mutation carriers show increased phase-based gamma connectivity and decreased amplitude-based beta connectivity

In human MEG data, wPLI was computed over frequencies ranging from 1 to 50 Hz. The resulting connectivity spectrum revealed a peak around 10 Hz (Fig. 5F). When analyzing functional connectivity for individual frequency bands, an increase in frontal-hippocampal connectivity was observed in mutation carriers in the gamma frequency band (Fig. 5G). No differences were found in frontal-parietal connectivity between mutation carriers and healthy controls. In contrast, several previous studies reproducibly indicated an increase in phase-based theta connectivity in symptomatic AD [53, 54]. The strongest effects were found in temporal regions in the theta band in AD dementia [53, 54, 66] and in frontal regions in the alpha band in MCI patients [67, 68]. Nevertheless, findings on regional or edge-level connectivity usually did not survive multiple comparisons correction and were not reproducible [53, 66, 68, 69].

Using the AECc, connectivity was highest within the alpha and beta bands (Fig. 6F). When quantifying connectivity in individual frequency bands, the beta 2 frequency band showed reduced functional connectivity in mutation carriers for both frontal-parietal and frontal-hippocampal connections (Fig. 6G). These results overlap with previously reported alterations in symptomatic AD [53, 54, 69].

## Discussion

To study the translational value of early AD-associated neurophysiological changes we measured oscillatory activity in 3–12-month-old APP/PS1 mice and in pre-symptomatic human subjects carrying autosomal-dominant AD mutations in the *APP* or *PSEN1* genes. We specifically compared changes in spectral characteristics and long-range functional connectivity. The main findings are discussed below and summarized in Table 2.

**Table 2:** Summary of network changes in APP/PS1 mice, in human pre-symptomatic and in human symptomatic AD

LFP/MEG parameter	APP/PS1 mice	Mutation carriers	Symptomatic AD^a^
**Total power**			
(Pre)frontal cortex	Unchanged	Unchanged	Unknown
Parietal cortex	Increased (3–6 moa)	Unchanged	Unknown
Hippocampus	Increased (3–6 moa)	Unchanged	Increased
**Absolute power**			
(Pre)frontal cortex	Unchanged	Unchanged	Increased *δ*, *θ*
Parietal cortex	Increased *θ*, *α*, *β*, *γ*1, *γ*2 (3–6 moa)	Unchanged	Increased *δ*, *θ,* in-/decreased *α*, decreased β
Hippocampus	Increased *δ*, *θ*, *α*, *β*, *γ*1, *γ*2 (3–6 moa)	unchanged	Increased *θ*, in-/decreased α, decreased *β*
**Relative power**			
(Pre)frontal cortex	Decreased *θ*, increased *β*, *γ*1 (3–12 moa)	Unchanged	Increased *θ*, increased α, decreased *β*
Parietal cortex	Decreased *θ*, increased *β*, *γ*1 (3–12 moa)	Unchanged	Increased *δ, θ*, in-/decreased *α*, decreased *β*
Hippocampus	Increased *β* (3–12 moa)	Increased θ	Increased *θ*, decreased α, *β*
**Peak frequency**			
Parietal cortex	Unchanged	Decreased α peak frequency	Decreased *α* peak frequency
Hippocampus	Unchanged	Decreased α peak frequency	Decreased *α* peak frequency
**Phase-based connectivity**			
(Pre)frontal-parietal	Decreased *θ*, *α* wPLI (6–12 moa)	Unchanged	Unchanged (suggested increased *θ*, in-/decreased *α*)
(Pre)frontal-hippocampal	Decreased *θ*, *α* wPLI (6–12 moa)	Increased *γ* wPLI	Unchanged (suggested increased *θ*)
**Amplitude-based connectivity**			
(Pre)frontal-parietal	Unchanged	Decreased *β* AECc	Decreased *α*, *β*
(Pre)frontal-hippocampal	Increased *β* AECc (9–12 moa)	Decreased *β* AECc	Decreased *α*, *β*

^a^For literature references, see the main text

### Higher absolute power in APP/PS1 mice but not in human *APP* and *PSEN1* mutation carriers

In APP/PS1 mice, absolute broadband power was transiently increased in the parietal cortex and hippocampus from approximately 3 to 6 moa. This increase was mainly due to higher absolute power in the beta and gamma bands. In line with these findings, several other studies also reported increased absolute power in mouse models of amyloid pathology. For example, absolute power was increased in most frequency bands in the medial frontal cortex of APP/PS1 mice at 5–6 moa [33] and in the posterior cortex at 7 moa [31]. In addition, hippocampal power was increased in the beta and gamma frequency bands in 4-month-old APP23 mice [70].

In contrast, pre-symptomatic human *APP* and *PSEN1* mutation carriers did not show alterations in total broad band or absolute power per frequency band. While absolute power has not yet been studied in pre-symptomatic mutation carriers to our knowledge, several MEG studies reported increased absolute power in early symptomatic sporadic AD. For instance, absolute power was increased in the theta and lower alpha bands in cortical regions as well as hippocampi of MCI and AD dementia patients [60, 62]. Another study showed increased global theta power and delta power in the left hemisphere in AD dementia patients [71]. Although caution should be taken when interpreting MEG-based absolute power due to high levels of inter-individual variability, absolute power might function as an indicator of neuronal activity [72, 73]. Increased neuronal firing has been linked to increased power [73, 74] and AD mouse models of amyloid pathology indeed exhibit increased numbers of hyperactive neurons [5–7]. This increase in neuronal activity is mediated by soluble amyloid beta [5, 6]. Taken together, increased absolute power, although in different frequency bands, seems a shared neurophysiological characteristic between mouse models of AD and symptomatic MCI and AD patients, which is however not yet observed in the pre-symptomatic *APP* and *PSEN1* mutation carriers included in the current study. It also should be noted that the early increase in absolute beta and gamma band power observed in APP/PS1 mice was transient in nature and disappeared at later ages, suggesting that it is not a good indicator of progressive cognitive decline.

### Opposite relative power shift between APP/PS1 mice and human *APP* and *PSEN1* mutation carriers

In APP/PS1 mice, we observed a shift in relative power from low frequencies (delta and theta) to high frequencies (beta and gamma). A similar shift towards higher frequencies has previously been reported in the frontal cortex, parietal cortex, and hippocampus of APP/PS1 mice [28, 29, 34, 75]. However, some studies also describe a shift towards lower frequencies, for example in the parietal cortex of J20 mice [76] and hippocampus of 5xFAD mice [77], and others did not find any alterations in relative power [78]. These conflicting findings may in part be explained by differences in behavioral state, type of AD animal model, or frequency range that is used for normalization.

In contrast to absolute power, alterations in relative power were stable over time (beta and gamma) or increased with age (theta), and thus may better reflect the progressive nature of cognitive dysfunction in mice. Interestingly, the increase in relative beta power was consistently observed in all three brain regions from the start of the measurements at 3 moa. Hippocampal beta oscillations have been implicated in novelty detection in mice [79–81]. Our findings thus suggest that increased beta oscillations precede and predict cognitive decline in mice.

In contrast to APP/PS1 mice, a significant increase in relative theta power was observed in the hippocampus of human pre-symptomatic *APP* and *PSEN1* mutation carriers. While similar neurophysiological alterations have been reported in the hippocampus of sporadic AD patients [35, 82] and in the precuneus of symptomatic *PSEN1* mutation carriers using source-modeled EEG [83], pre-symptomatic *PSEN1* mutation carriers showed a decrease in relative theta power and an increase in alpha2 power in the precuneus [83, 84]. These dissimilarities between previous and current findings may largely be explained by differences in age (29 resp. 49 years), modality (EEG resp. MEG), or methodology (ICA resp. source space MEG using beamformer).

Peak frequency in mice was characterized around 8 Hz and was unaltered in APP/PS1 mice except for a possible trend towards reduction at 3 moa in awake moving animals. Several studies have reported a decrease in peak frequency in AD mouse models [85–87] while others found no change [85, 87–89]. The resting-state alpha peak in human *APP* and *PSEN1* mutation carriers was characterized at around 9 Hz and was decreased in the parietal cortex and the hippocampus, consistent with oscillatory slowing in AD [82, 90, 91].

### Different functional connectivity alterations between APP/PS1 mice and human *APP* and *PSEN1* mutation carriers

Using a phase-based connectivity measure (wPLI), APP/PS1 mice exhibited reduced long-range connectivity in the theta/alpha range, which became more pronounced with age, whereas amplitude-based connectivity (AECc) revealed increased connectivity in the beta frequency band. Few studies have investigated connectivity using electrophysiological recordings in AD mice. In APP/PS1 mice, phase coherence between the perforant path and dentate gyrus was decreased in the theta, alpha, and gamma frequency ranges [92], and delta connectivity was decreased between the frontal cortex and CA1 during sleep [33].

In contrast, human *APP* and *PSEN1* mutation carriers show increased phase-based gamma connectivity between the frontal cortex and hippocampus and decreased amplitude-based beta connectivity for both frontal-parietal and frontal-hippocampal connections. A recent MEG study in amnestic MCI patients with subclinical epileptiform activity showed decreased phase-based gamma connectivity instead [93]. The increase in gamma wPLI observed in the current study may thus point to a pre-symptomatic stage that precedes neuronal hyperexcitability. In line with the reduced amplitude-based beta connectivity observed here, a number of fMRI studies reported decreased connectivity in human *PSEN1* mutation carriers, specifically in the default mode network [38, 94, 95].

Despite these potentially interesting results, it needs to be stressed that alterations in connectivity in APP/PS1 mice and human mutation carriers do not overlap. Also, opposite directions are observed between amplitude- and phase-based connectivity changes, which may reflect differences in modes of functional connectivity that may complement each other [53, 96, 97]. Whether or not connectivity changes reflect neuronal hyperexcitability might be answered using computational modeling.

### Translating neurophysiological alterations between mice and humans

The current study is to our knowledge the first to directly compare neurophysiological changes between an APP/PS1 mouse model and pre-symptomatic human *APP* and *PSEN1* mutation carriers. Changes in asymptomatic mutation carriers, reflecting a very early stage of AD before subjective complaints develop, do not seem to uniformly correspond to any specific time point along the 9-month period during which we performed LFP recordings in mice. Instead, some LFP features in APP/PS1 mice seem to match later stages of AD reported in the literature. Several factors may underlie these apparent discrepancies.

Firstly, AD mouse models may not exactly recapitulate the time course of human disease. APP/PS1 mice, for example, exhibit APP overexpression from an early age, whereas in humans there is a far more gradual increase in amyloid levels. Indeed, APP knock-in mice that more gradually develop amyloidosis without APP overexpression showed no oscillatory power changes at 3 moa [98–100] and impaired gamma oscillations only at 6 moa [101]. Despite this mismatch between mice and humans, some of our findings may hint towards disease-relevant mechanisms. For instance, the progressive decrease in relative theta power and connectivity reflects cognitive decline in mice. In contrast, some changes in APP/PS1 mice are increased from the start of the recording period (e.g., relative beta and gamma power), suggesting a link with soluble amyloid, while others are transiently increased from 3–6 months only (e.g., total, alpha, beta and low gamma power), suggesting a link with the onset of amyloid plaque deposition. These observations are potentially clinically relevant and should be further investigated.

Secondly, many AD mouse models do not show neurodegeneration or tau pathology [102], which are characteristic of the human disease. Interestingly, several reports have indicated that tauopathy can attenuate neuronal excitability [103–106] and the combination of amyloid and tau pathology can increase the number of silent neurons [104]. In line with these findings, a recent neurophysiological study in a combined amyloid and tau mouse model showed decreased gamma power compared to amyloid-only models [98], which fits recent data indicating decreased frontal gamma activity in human AD dementia patients [9]. The increase in absolute gamma power in transgenic mouse models with amyloid pathology only may thus be the combined result of overexpression of amyloid and lack of tau pathology.

Finally, the inconsistent effects on relative theta power in APP/PS1 mice and human mutation carriers may be explained by differences in cholinergic function. In AD dementia patients and individuals with MCI, the general and progressive slowing of oscillatory activity [2, 35] has been associated with decreased functioning of the cholinergic system [107]. Reducing acetylcholine availability using the muscarinic acetylcholine receptor antagonist scopolamine enhances slow wave activity in AD patients [108], while oscillatory slowing is counteracted by acetyl-cholinesterase inhibitors that increase the availability of acetylcholine [109–111]. Similarly, in mice slow wave power can be enhanced or decreased using chemogenetic inhibition or activation, respectively, of cholinergic neurons in the basal forebrain [112]. Enhanced theta power in AD may thus depend on the extent of cholinergic degeneration, which is prominent in AD patients, but detected only at later ages in some mouse models of AD (for review, see [113]). It will be of interest to investigate the link between cholinergic degeneration and (the absence of) oscillatory slowing in AD mouse models in future studies.

### Limitations and future directions

Even though we compared mice and humans with mutations in the same genes (*APP* and *PSEN1*), there are several limitations in comparing our results. First, the spectral power and peak frequencies possibly reflect different neurophysiological mechanisms in mice and humans. For example, whereas theta peak frequency in mice is positively modulated by speed, alpha peak frequency in humans is most prominent during rest [114, 115]. Further research will be needed to clarify the relationship between oscillatory frequencies in mice and humans as well as their potential value as biomarkers of pre-symptomatic AD. Second, even though we only included human subjects with proven *APP* or *PSEN1* mutations and no cognitive impairment, subjects may have been in different pre-symptomatic stages of the disease. Although symptom onset is claimed to be rather predictable within families [116], disease staging is difficult and three of our mutation carriers were even 6 years or more beyond their predicted age of symptom onset, suggesting that they may have been atypical. Third, the mutations themselves are not the same in APP/PS1 mice and human mutation carriers, which potentially results in differences in amyloid protein configuration and its effects on local oscillatory activity and functional connectivity. Fourth, LFP and MEG recordings are different in nature. Arguably, EEG signals would be more similar to LFP signals in mice, however, MEG has been reported to measure similar spectral and functional connectivity changes as EEG in early and later stages of AD and carries the advantage of allowing for higher spatial resolution as well as better source reconstruction of signals in deep brain structures such as the hippocampus. Nevertheless, comparison of high-density EEG recordings in mice [117], potentially in combination with LFP recordings, with E/MEG recordings in humans will be useful in the future. Last, the alignment of behavioral states between mice and humans is challenging as mice cannot be instructed to rest with eyes closed and human MEG recordings during free movement are not yet possible, although the development of wearable MEG systems will open up new possibilities [118]. In addition, all recordings were performed in the absence of a cognitive challenge, which may have masked genotypic effects. It will be of interest to further explore genotypic neurophysiological signatures in relation to memory performance in both mice and humans.

## Conclusions

Neurophysiological measurements form an important translational bridge between potential disease mechanisms discovered in mice and the pathophysiological progression of AD in humans. In this study, we found discordant changes in spectral characteristics and functional connectivity in APP/PS1 mice and human pre-symptomatic *APP* and *PSEN1* mutation carriers, raising awareness regarding the direct translatability of neurophysiological findings in mouse models. At the same time, we provide a starting point for future endeavors to bridge the neurophysiological gap between AD mice and patients by further improving the face validity of mouse models of AD and harmonizing the way in which measurements are acquired in both species.

### Supplementary Information

**Additional file 1:** **Supplementary Methods. Supplementary Figure 1.** LFP electrode coordinates.** Supplementary Figure 2.** Classification of hippocampal electrodes. **Supplementary Figure 3.** Assigning behavioral states to LFP recordings. **Supplementary Figure 4.** Between-MEG scanner variability. **Supplementary Figure 5.** Characterization of behavioral states in mice. **Supplementary Figure 6.** AD-related changes in absolute power in APP/PS1 mice during quiet wake. **Supplementary Figure 7.** AD-related changes in relative power in APP/PS1 during quiet wake. **Supplementary Figure 8.** AD-related changes in theta peak frequency in APP/PS1 mice during quiet wake. **Supplementary Figure 9.** AD-related changes in phase-based connectivity, as measured by the weighted phase lag index (wPLI), in APP/PS1 mice during quiet wake. **Supplementary Figure 10.** AD-related changes in amplitude-based connectivity, as measured by amplitude envelope correlation (AEC), in APP/PS1 mice during quiet wake. **Supplementary Table 1.** APP and PSEN1 mutations of pre-symptomatic human mutation carriers. **Supplementary Table 2.** Subdivision of regions of interest (ROIs) as in the automatic anatomical labeling atlas (AAL). **Supplementary Table 3.** Mixed-effects analysis of velocity. **Supplementary Table 4.** Neuropsychological, subjective cognitive decline and psychiatric test scores. **Supplementary Table 5.** Mixed-effects analysis of absolute power. **Supplementary Table 6.** Mixed-effects analysis of relative power. **Supplementary Table 7.** Mixed-effects analysis of theta peak frequency. **Supplementary Table 8.** Mixed-effects analysis of wPLI. **Supplementary Table 9.** Mixed-effects analysis of AECc.

## Data Availability

All data are available from the corresponding author upon reasonable request.
