# Supplementary material for: Neurophysiological alterations in mice and humans carrying mutations in APP and PSEN1 genes
Source: Alzheimers Res Ther. 2023 Aug 22;15:142. doi: 10.1186/s13195-023-01287-6 (PMC10464047; doi:10.1186/s13195-023-01287-6)
Supplement: Supplementary file 1 — Additional file 1: Supplementary Methods. Supplementary Figure 1. LFP electrode coordinates. Supplementary Figure 2. Classification of hippocampal electrodes. Supplementary Figure 3. Assigning behavioral states to LFP recordings. Supplementary Figure 4. Between-MEG scanner variability. Supplementary Figure 5. Characterization of behavioral states in mice. Supplementary Figure 6. AD-related changes in absolute power in APP/PS1 mice during quiet wake. Supplementary Figure 7. AD-related changes in relative power in APP/PS1 during quiet wake. Supplementary Figure 8. AD-related changes in theta peak frequency in APP/PS1 mice during quiet wake. Supplementary Figure 9. AD-related changes in phase-based connectivity, as measured by the weighted phase lag index (wPLI), in APP/PS1 mice during quiet wake. Supplementary Figure 10. AD-related changes in amplitude-based connectivity, as measured by amplitude envelope correlation (AEC), in APP/PS1 mice during quiet wake. Supplementary Table 1. APP and PSEN1 mutations of pre-symptomatic human mutation carriers. Supplementary Table 2. Subdivision of regions of interest (ROIs) as in the automatic anatomical labeling atlas (AAL). Supplementary Table 3. Mixed-effects analysis of velocity. Supplementary Table 4. Neuropsychological, subjective cognitive decline and psychiatric test scores. Supplementary Table 5. Mixed-effects analysis of absolute power. Supplementary Table 6. Mixed-effects analysis of relative power. Supplementary Table 7. Mixed-effects analysis of theta peak frequency. Supplementary Table 8. Mixed-effects analysis of wPLI. Supplementary Table 9. Mixed-effects analysis of AECc. [file 13195_2023_1287_MOESM1_ESM.pdf]

SUPPLEMENTARY METHODS

Selection of hippocampal electrodes

Hippocampal electrodes were grouped based on their location relative to the pyramidal layer: supra-pyramidal, pyramidal and infra-pyramidal. The location of an electrode was determined based on ripple amplitude and theta phase (Supplementary Fig. 2). First, ripples were manually searched for in one of the first three and one of the last three recordings of each animal. Using MATLAB, recordings were bandpass-filtered between 150 Hz and 250 Hz and the identified time window selected. The two electrodes (one from each row of hippocampal electrodes) with the largest ripple amplitude were identified as ‘pyramidal’. If other electrodes had similar ripple amplitudes, these were also categorized as ‘pyramidal’. In case ripples were not found, no electrodes were labeled ‘pyramidal’. Next, theta (5-10 Hz) phase was computed for all electrodes. As theta phase reverses below the pyramidal layer[1], electrodes with a phase similar to the parietal electrodes were classified as ‘supra-pyramidal’ and electrodes with a different theta phase different from the parietal electrodes were classified as ‘infra-pyramidal’. Subsequently, electrode classifications of the first and last recordings were compared as electrodes might have moved over the course of the 9-month recording period. If the location of an electrode did not change during the experiment, it retained its label. In case the electrode picking up the largest ripple amplitude changed, both electrodes were labeled ‘pyramidal’. If these two electrodes were not adjacent, the electrodes in between were also classified as ‘pyramidal’ (max. 3 ‘pyramidal’ electrodes per row of hippocampal electrodes). If the pyramidal electrodes shifted more than two electrodes to the side, no electrodes were labeled as ‘pyramidal’. ‘Supra-pyramidal’ and ‘infra-pyramidal’ electrodes were only classified as such when they had retained their location during the entire recording period.

MEG preprocessing

An atlas-based beamforming approach[2] was used to obtain source-level MEG time series. In short, each voxel of the co-registered MRI was labeled to one of the 90 regions of the AAL-atlas. The timeseries of each voxel was reconstructed using projection of sensor level MEG data through normalized beamformer weights and using an equivalent current dipole volume conductor model.[3] The MEG signal of the centroid voxel of each region was used as representative for that region.[4] The source-reconstructed MEG time series were converted to ASCII files and imported into an in-house developed software package, BrainWave, a Java based application for visualization (https://home.kpn.nl/stam7883/ brainwave.html).

Neuropsychological examination

The MMSE was used as a global index of cognitive performance.[5] The following cognitive domains were evaluated: memory (Visual Association Test, Dutch version of Rey Auditory Verbal Learning Test, Rey Complex Figure Recall Task), attention (Digit span forward, Trail Making Test (TMT) A, Stroop color word test I and II), executive functioning (TMT-B, Digit span backwards, Stroop color word test III, letter fluency test (version D-A-T)), language (category animal fluency, visual association test – 'naming') and visuo-spatial functioning (Rey Complex Figure Copy Task, Visual Object and Space Perception Battery (VOSP) (number location)). Raw test scores were adjusted for age, sex and education and converted to *t*-scores or percentiles.[6] A *t*-score of 36 means that the subject's score is 1.4 standard deviation below the expected score and that 9% of the people with similar age, sex and education performed worse than the studied subject. *T*-scores of 36 or lower were considered abnormal.

For two mutation carriers neuropsychological data was available from the SCIENCe study cohort (2014.019) of the Amsterdam UMC (location VUmc) for which all previously mentioned but The Rey Complex Figure Tasks, the Digit span forward and backwards and the VOSP were examined. Ten mutation carriers received cognitive and clinical examination within 2 months before or after MEG recording. One mutation carrier had clinical and neuropsychological data available from 14 months prior to MEG recording through the SCIENCe study.

Subjective cognitive assessment

Self-reported cognitive decline of mutation carriers was assessed through two questionnaires. Subjective cognitive function compared to 1 and 5 years earlier was assessed with the Subjective Cognitive Functioning (SCF) questionnaire[7] and the Dutch translation of the Cognitive Change Index (CCI),[8] respectively. Psychiatric symptoms were evaluated using the Hospital Anxiety and Depression Scale (HADS).[9] An informant of each participant completed a short version of the Amsterdam questionnaire measuring "instrumental activities of daily living" (IADL) in (early) dementia[10]. The SCF questionnaire for informants was also obtained to assess informant reported decline over a 1-year period.

The SCF questionnaire consists of 4 questions (range -12 to +12).[7] SCF scores <0 represent self or informant reported cognitive decline. The CCI consists of 20 questions (range 20-100).[8] Higher CCI scores reflect worse cognitive function (> 40: mild changes). The HADS is subdivided in 7 items about depression and 7 items about anxiety (range 0 – 21 for each).[9] Higher (sub)scores reflect more psychiatric complaints (0-7: no anxiety or depressive symptoms, 8-10: possible anxiety or depression, 11-21: suspected anxiety disorder or depression). The IADL in (early) dementia was converted to *t*-

scores using the Item Response Theory[11] and measures in a range of 20-80. Lower total IADL scores reflect more severe functional impairments and scores below 51.4 were considered abnormal.[12]

References

1. Scheffer-Teixeira R, Belchior H, Caixeta FV, Souza BC, Ribeiro S, Tort AB. Theta phase modulates multiple layer-specific oscillations in the CA1 region. *Cereb Cortex*. Oct 2012;22(10):2404-14.

2. Hillebrand A, Barnes GR, Bosboom JL, Berendse HW, Stam CJ. Frequency-dependent functional connectivity within resting-state networks: an atlas-based MEG beamformer solution. *NeuroImage*. Feb 15 2012;59(4):3909-21.

3. Cheyne D, Bostan AC, Gaetz W, Pang EW. Event-related beamforming: a robust method for presurgical functional mapping using MEG. *Clin Neurophysiol*. Aug 2007;118(8):1691-704.

4. Hillebrand A, Tewarie P, van Dellen E*, et al*. Direction of information flow in large-scale resting-state networks is frequency-dependent. *Proc Natl Acad Sci U S A*. Apr 05 2016;113(14):3867-72.

5. Folstein MF, Folstein SE, McHugh PR. "Mini-mental state". A practical method for grading the cognitive state of patients for the clinician. *J Psychiatr Res*. Nov 1975;12(3):189-98.

6. Schmand B. HP, de Koning I. Normen van psychologische tests voor gebruik in de klinische neuropsychologie. Nederlands Instituut van Psychologen. https://www.psynip.nl/secties/sectie-neuropsychologie/

7. Aalten P, Ramakers IH, Biessels GJ*, et al*. The Dutch Parelsnoer Institute--Neurodegenerative diseases; methods, design and baseline results. *Bmc Neurol*. Dec 31 2014;14:254.

8. Rattanabannakit C, Risacher SL, Gao S*, et al*. The Cognitive Change Index as a Measure of Self and Informant Perception of Cognitive Decline: Relation to Neuropsychological Tests. *J Alzheimers Dis*. 2016;51(4):1145-55.

9. Spinhoven P, Ormel J, Sloekers PP, Kempen GI, Speckens AE, Van Hemert AM. A validation study of the Hospital Anxiety and Depression Scale (HADS) in different groups of Dutch subjects. *Psychol Med*. Mar 1997;27(2):363-70.

10. Sikkes SA, Knol DL, Pijnenburg YA, de Lange-de Klerk ES, Uitdehaag BM, Scheltens P. Validation of the Amsterdam IADL Questionnaire(c), a new tool to measure instrumental activities of daily living in dementia. *Neuroepidemiology*. 2013;41(1):35-41.

11. Reise SP, Waller NG. Item response theory and clinical measurement. *Annu Rev Clin Psychol*. 2009;5:27-48.

12. Sikkes SA, Pijnenburg YA, Knol DL, de Lange-de Klerk ES, Scheltens P, Uitdehaag BM. Assessment of instrumental activities of daily living in dementia: diagnostic value of the Amsterdam Instrumental Activities of Daily Living Questionnaire. *J Geriatr Psychiatry Neurol*. Dec 2013;26(4):244-50.

SUPPLEMENTARY FIGURES

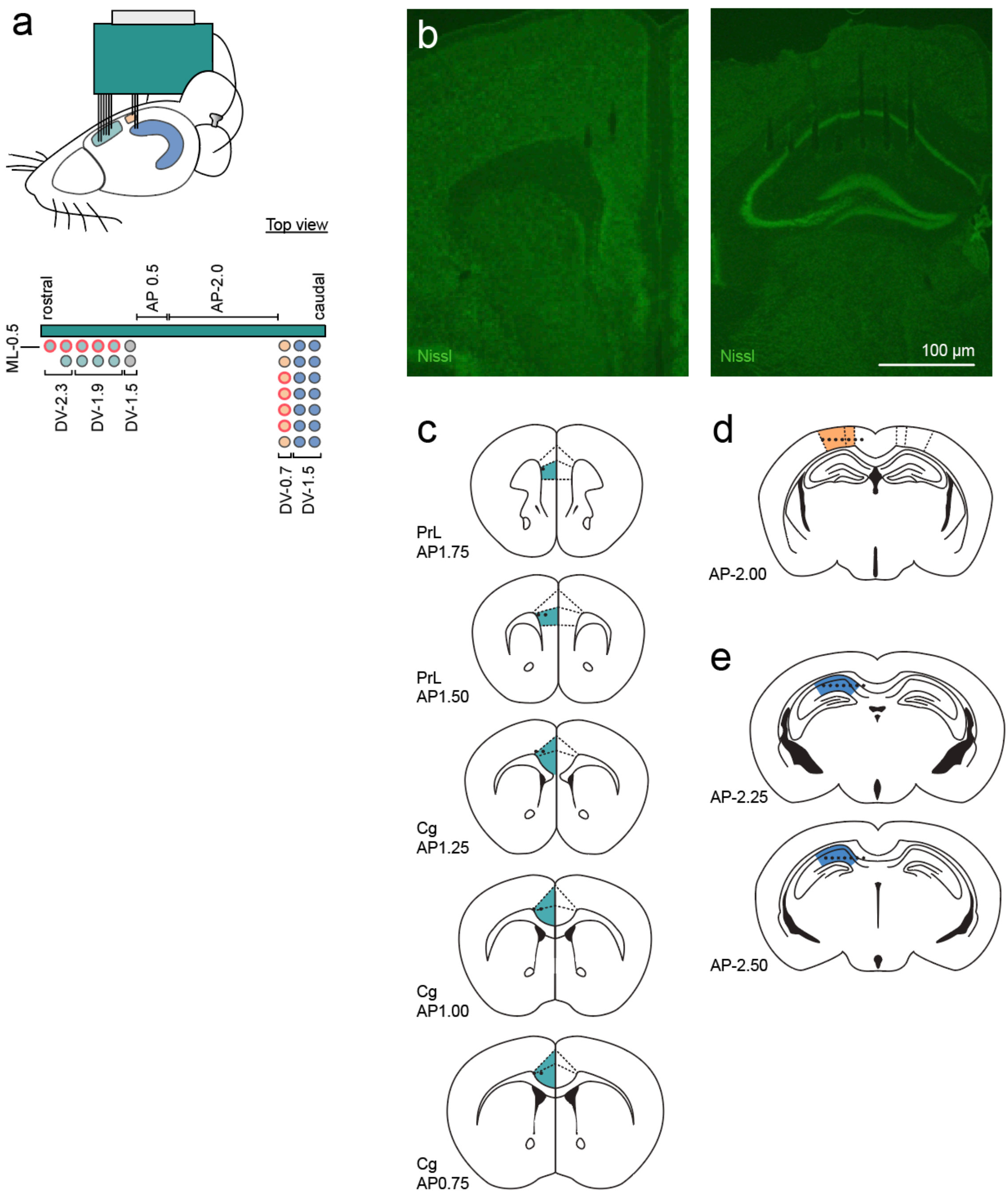

Supplementary Figure 1. LFP electrode coordinates.
(A) Graphical representation of electrodes used for LFP recordings. Electrodes were placed in prefrontal cortex (light green), parietal cortex (orange) and hippocampus (blue). A reference screw was located at the cerebellum. Electrodes were connected to a circuit board (dark green rectangle). Bottom: Top view of electrode arrays. Prefrontal (green) and parietal (orange) electrodes that were used for analysis are outlined in red. Selection of hippocampal electrodes for analysis was based on LFP traces (see Fig. S4).
(B) Example microscope images of electrodes in prefrontal cortex (left) and in hippocampus (right). Coronal sections were incubated with green fluorescent Nissl staining.
(C-E) Coordinates of prefrontal (C), parietal (D) and hippocampal (E) electrodes. Coronal sections are based on the Allen mouse brain atlas.

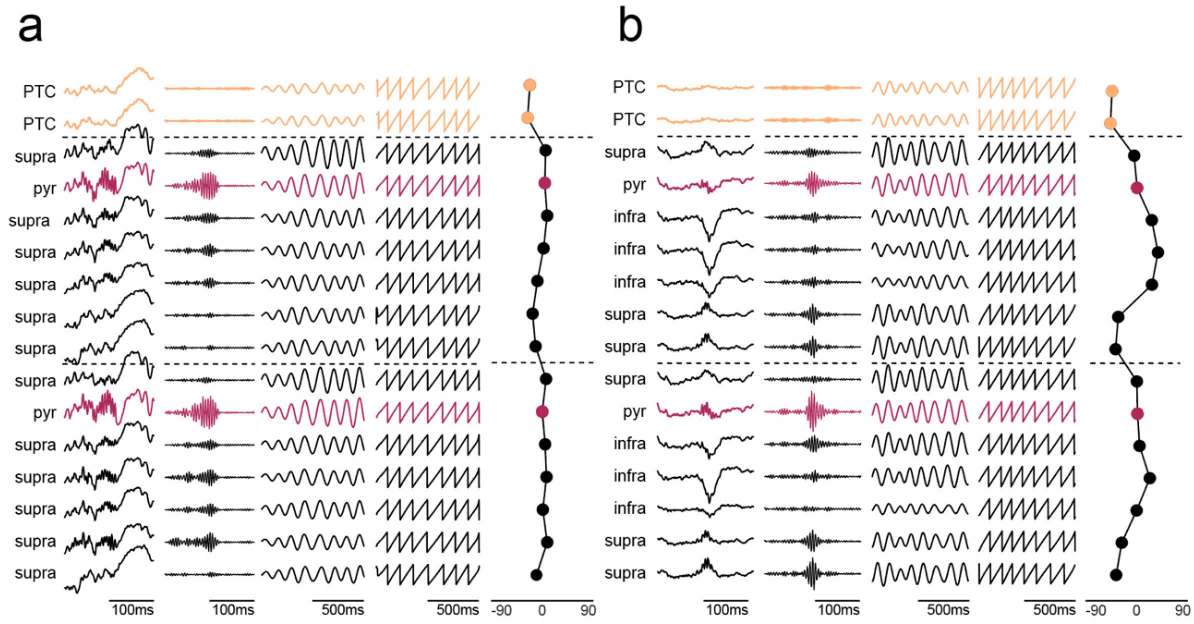

Supplementary Figure 2. Classification of hippocampal electrodes.
Example electrode classification in a wild-type (A) and APP/PS1 (B) animal. The first column depicts raw LFP data. In the second column filtered (150-250 Hz) data are plotted. The channels with maximum ripple amplitude are colored in red. The third column contains theta-filtered (5-10 Hz) data. Theta phase, and theta phase respective to the pyramidal layer, are plotted in the fourth and fifth column. Channels are classified as supra-pyramidal, pyramidal and infra-pyramidal based on ripple amplitude and theta phase.

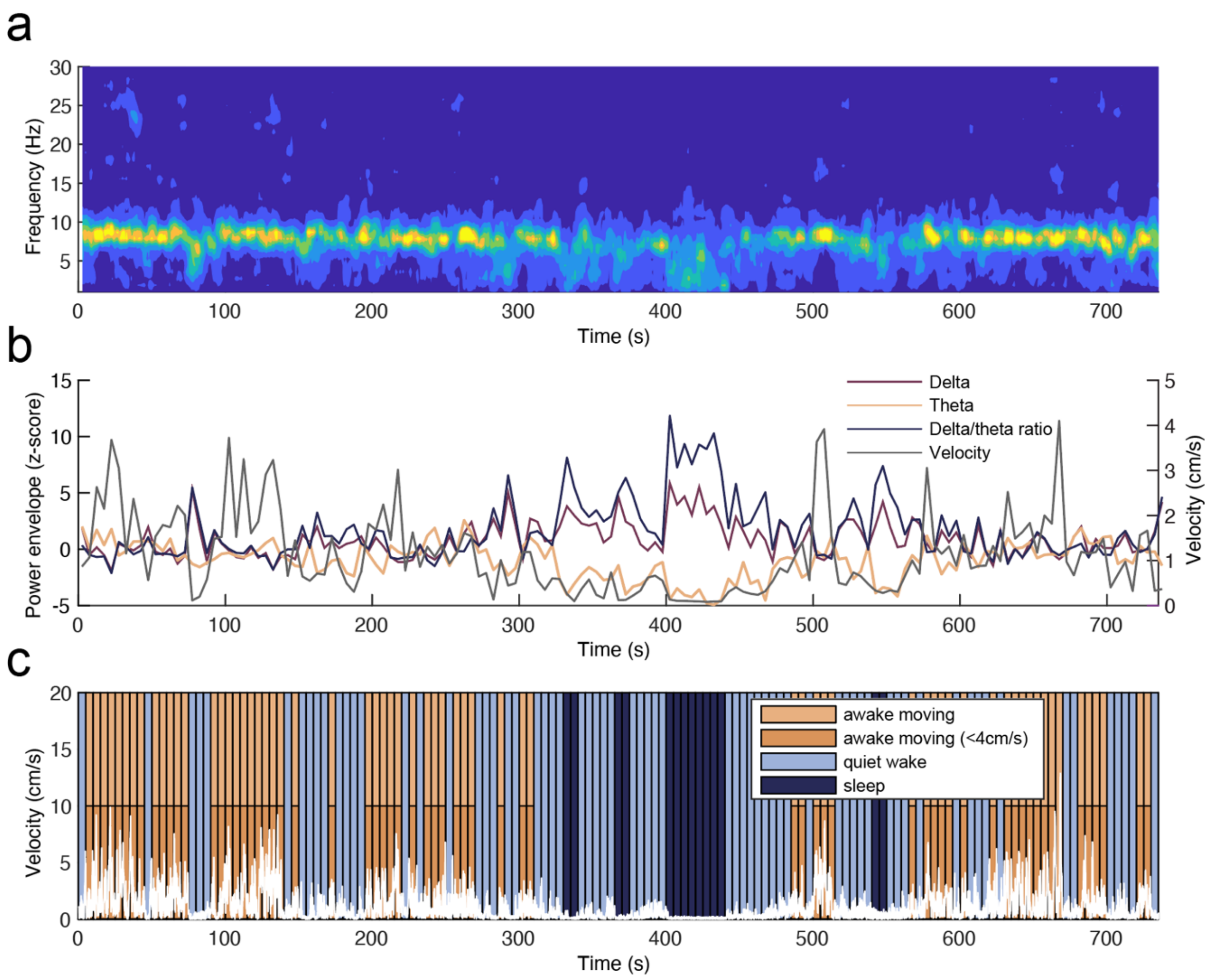

Supplementary Figure 3. Assigning behavioral states to LFP recordings.
(A) Example spectrogram.
(B) Mean delta envelope, theta envelope, delta/theta ratio (left y-axis) and velocity (right y-axis) were calculated for 5-s epochs of the LFP recording shown in (A).
(C) Behavioral states (awake moving, quiet wake and sleep) were assigned to 5-s epochs based on the delta/theta ratio and velocity. For the analysis of awake mobility, epochs were selected in which the average velocity did not exceed 4cm/s. Overlaying the behavioral epochs, raw velocity that was calculated based on the video frames has been plotted in white.

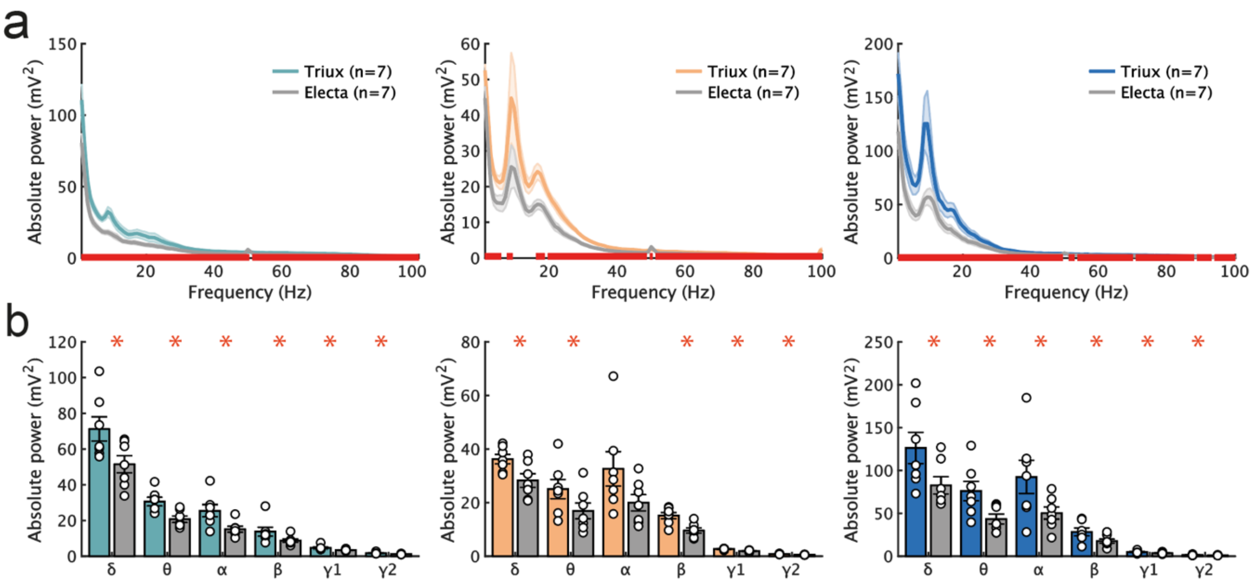

Supplementary Figure 4. Between-MEG scanner variability. MEG-scanner related changes in absolute power measures in human subjects. Healthy subjects (n=7) underwent resting-state eyes-closed MEG recording on both the Electa and the novel Triux neo MEG system. The median difference in time between scans was 5.9 years (IQR = 5.3 – 9.4). Groups significantly differed in age (median age Electa: 47 years (IQR = 38 – 50), Triux neo: 52 years (IQR = 43 – 59, W=45, p=0.004). Raw data was downsampled to 250 Hz. Ten epochs of ~16 sec each were selected for further analysis. Significant differences between scanners using non-parametric Wilcoxon signed-rank test are referred to in the figure with a red bar on the x-axis, or a red asterisk.

(A) Mean absolute PSD in frontal cortex (green), parietal cortex (orange) and hippocampus (blue) of the left hemisphere.

(B) Quantification of absolute power per frequency band, from left to right: delta (1-4 Hz), theta (4-8 Hz), alpha (8-13 Hz), beta (13-30 Hz), low gamma (30-60 Hz) and high gamma (60-120 Hz). Absolute power was higher for Triux data in frontal cortex (δ: W=27.0, p=0.031; θ: W=28.0, p=0.016, α: W=28.0, p=0.016; β: W=28.0, p=0.016; γ1: W=28.0, p=0.016; γ2: W=28.0, p=0.016), parietal cortex (δ: W=28.0, p=0.016; θ: W=27.0, p=0.031, α: W=25, p=0.078; β: W=28.0, p=0.016; γ1: W=28.0, p=0.016; γ2: W=28.0, p=0.016), and hippocampus (δ: W=28.0, p=0.016; θ: W=28.0, p=0.016, α: W=28.0, p=0.016; β: W=28.0, p=0.016; γ1: W=28.0, p=0.016; γ2: W=28.0, p=0.016).

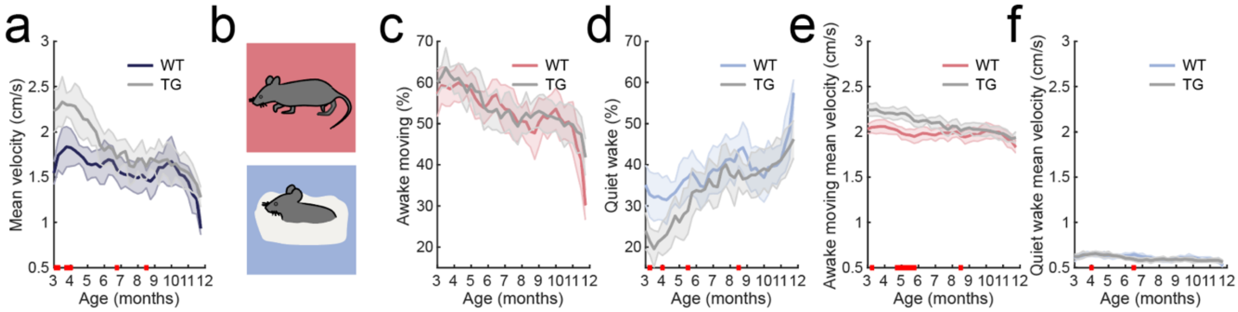

Supplementary Figure 5. Characterization of behavioral states in mice. Results of mixed-effects analysis are described in the legend. Significant results from uncorrected two-sample tests are depicted by a red bar on the x-axis.
(A) Mean velocity is not significantly different between APP/PS1 and wildtype mice.
(B) Recordings were separated into three behavioral states: awake moving (top), quiet wake (bottom) and sleep. Sleep epochs were excluded from analysis.
(C) The percentage of time spent moving is not significantly different between APP/PS1 and wildtype mice.
(D) The percentage of time spent in quiet wake is not significantly different between APP/PS1 and wildtype mice. However, at 3-4 moa, APP/PS1 mice seem to spend less time in quiet wake than wildtype mice.
(E) Mean velocity during movement epochs is not significantly different between genotypes.
(F) Mean velocity during quiet wake epochs is not significantly different between genotypes.

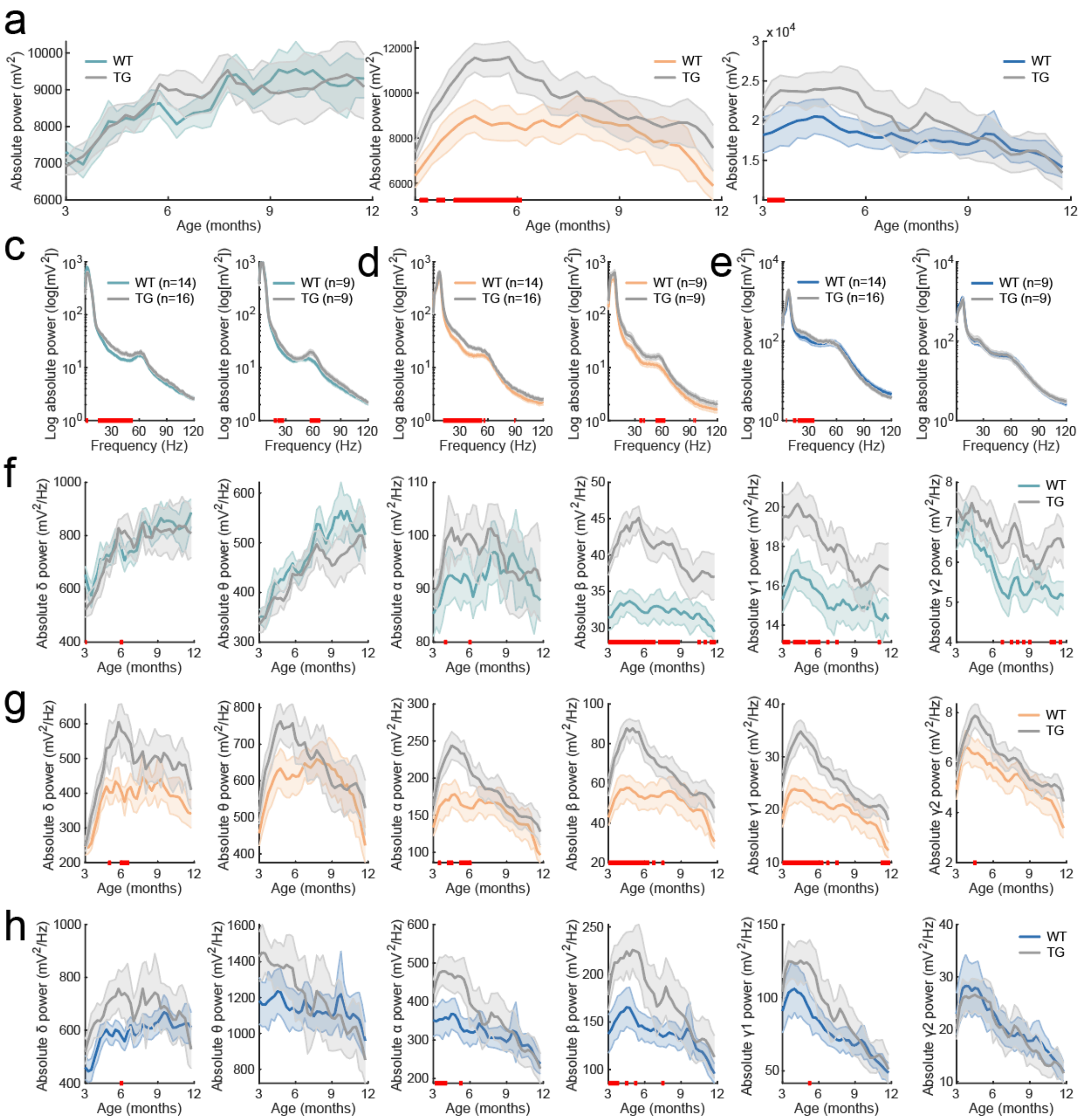

Supplementary Figure 6. AD-related changes in absolute power in APP/PS1 mice during quiet wake. Results of mixed-effects analysis are referred to in legends: ^ main effect of genotype; * interaction effect genotype x time. Significant results from uncorrected two-sample tests are depicted by a red bar on the x-axis.

(A) Total absolute power (absolute power summed over 1-120 Hz) in prefrontal cortex (left), parietal cortex (middle) and hippocampus (right) in 3-12-month-old APP/PS1 and wildtype mice. Parietal cortex: *; hippocampus: *

(B) Mean PSD for 3-month-old (left) and 12-month-old (right) mice in prefrontal cortex.

(C) Mean PSD for 3-month-old (left) and 12-month-old (right) mice in parietal cortex.

(D) Mean PSD for 3-month-old (left) and 12-month-old (right) mice in hippocampus.

(E) Mean absolute power per frequency band over the 9-month recording period in prefrontal cortex. Frequency bands from left to right: delta (1-5 Hz), theta (5-10 Hz), alpha (10-13 Hz), beta (13-30 Hz), low gamma (30-60 Hz) and high gamma (60-120 Hz). β: ^*, γ1: *

(F) Mean absolute power per frequency band over the 9-month recording period in parietal cortex. α: *, β: *, γ1: *

(G) Mean absolute power per frequency band over the 9-month recording period in hippocampus. θ: *, α: *, β: *, γ1: *, γ2: *

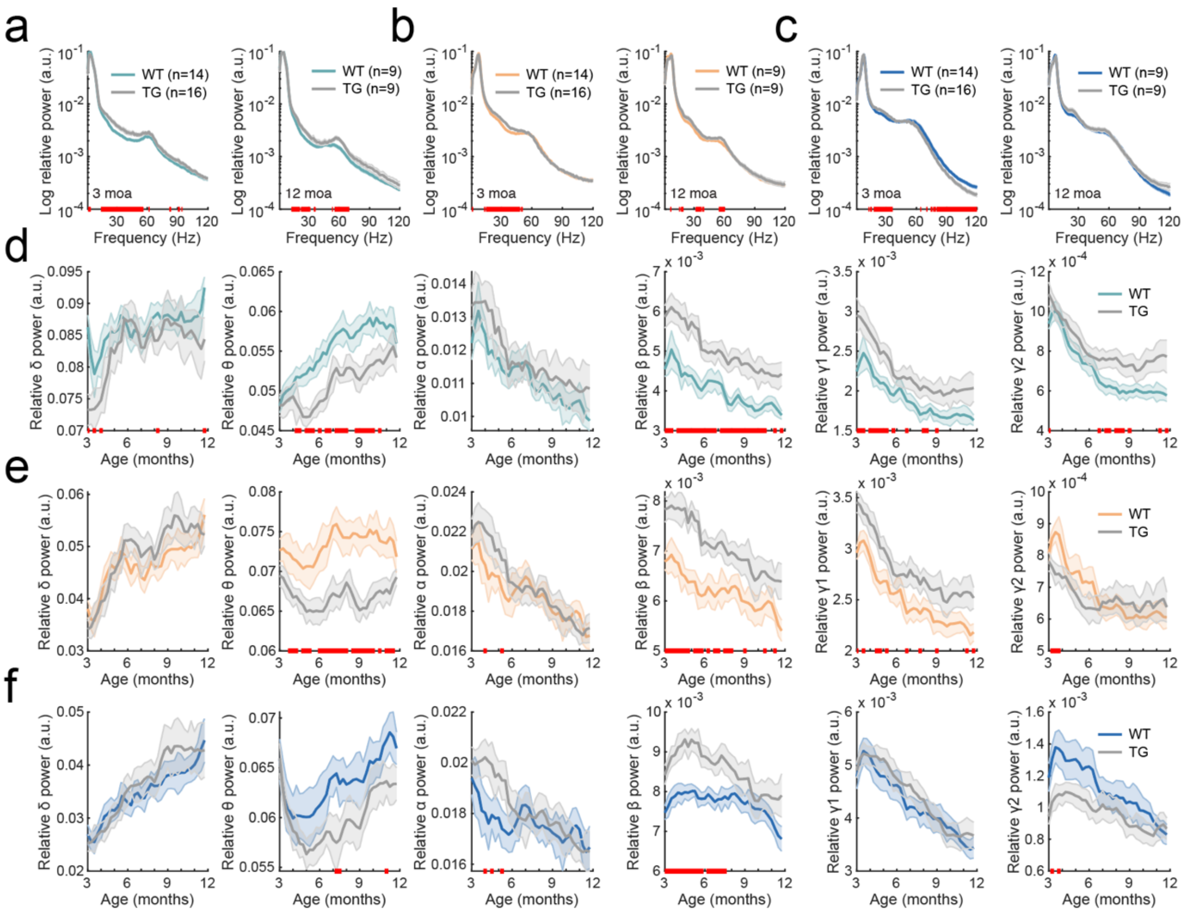

Supplementary Figure 7. AD-related changes in relative power in APP/PS1 during quiet wake. Results of mixed-effects analysis are referred to in legends: ^ main effect of genotype; * interaction effect genotype x time. Significant results from uncorrected two-sample tests are depicted by a red bar on the x-axis.

(A-C) Mean PSD for 3-month-old (left) and 12-month-old (right) mice in prefrontal cortex (A), parietal cortex (B) and hippocampus (C).

(D) Mean relative power per frequency band over the 9-month recording period in prefrontal cortex. Frequency bands from left to right: delta (1-5 Hz), theta (5-10 Hz), alpha (10-13 Hz), beta (13-30 Hz), low gamma (30-60 Hz) and high gamma (60-120 Hz). δ: *, θ: ^; β: ^*, γ1: *

(E) Mean relative power per frequency band over the 9-month recording period in parietal cortex. θ: ^; β: ^*, γ1: ^, γ2: *

(F) Mean relative power per frequency band over the 9-month recording period in hippocampus. β: ^*, γ1: *, γ2: ^

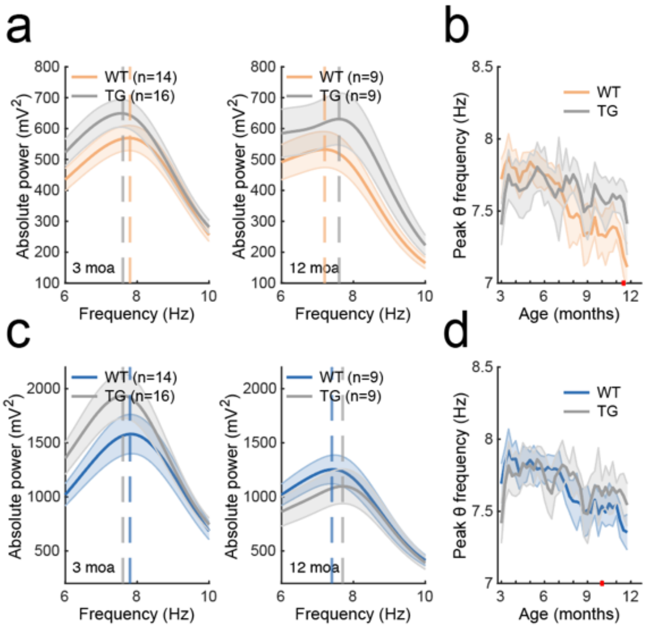

Supplementary Figure 8. AD-related changes in theta peak frequency in APP/PS1 mice during quiet wake. Results of mixed-effects analysis are referred to in legends: ^ main effect of genotype; * interaction effect genotype x time. Significant results from uncorrected two-sample tests are depicted by a red bar on the x-axis.

(A) Mean PSD for theta frequencies over which peak frequency was calculated (6-10 Hz) in 3-month-old (left) and 12-month-old (right) mice in parietal cortex.

(B) Mean theta peak frequency over the 9-month recording period in parietal cortex. Mixed-effects analysis did not show a main effect of genotype or a genotype x time interaction effect.

(C) Mean PSD for theta frequencies in 3-month-old (left) and 12-month-old (right) mice in hippocampus.

(D) Mean theta peak frequency over the 9-month recording period in hippocampus. Mixed-effects analysis did not show a main effect of genotype or a genotype x time interaction effect.

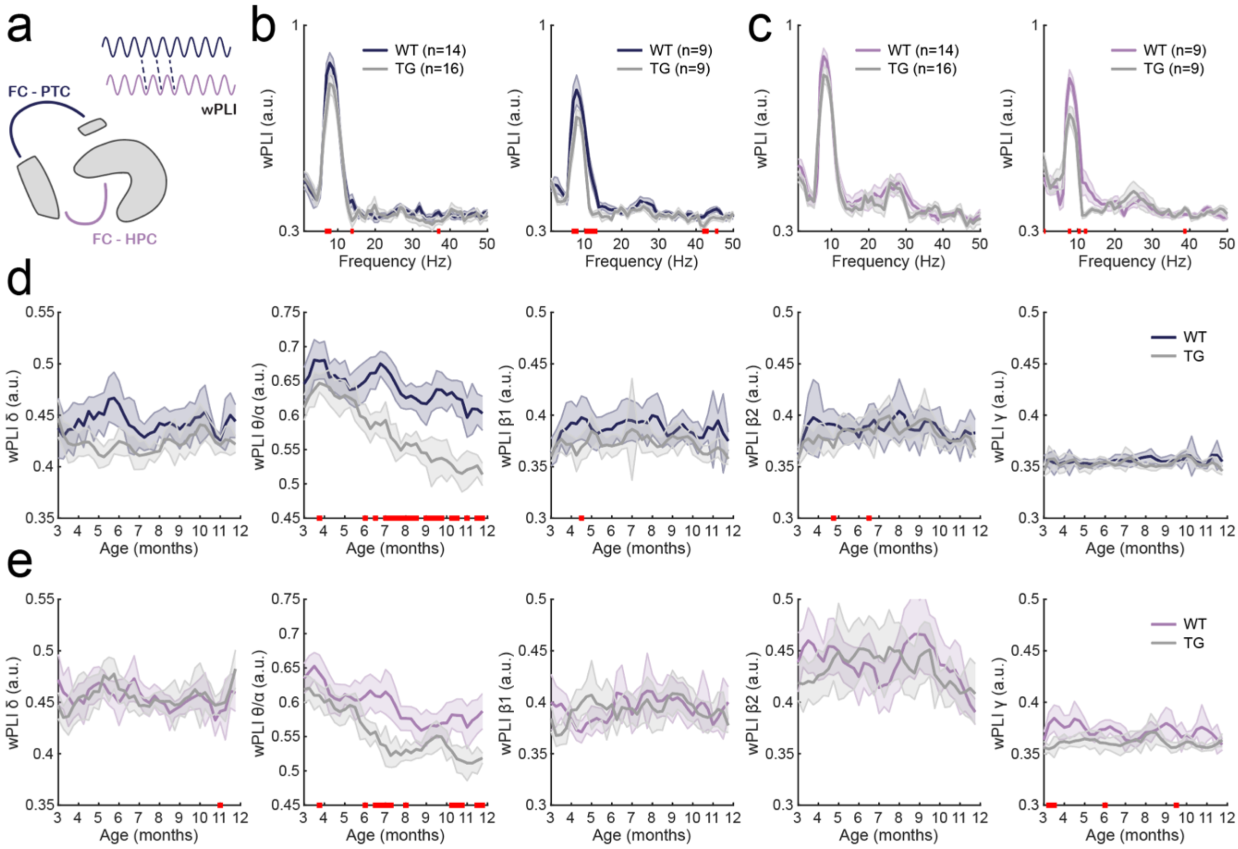

Supplementary Figure 9. AD-related changes in phase-based connectivity, as measured by the weighted phase lag index (wPLI), in APP/PS1 mice during quiet wake. Results of mixed-effects analysis are referred to in legends: ^ main effect of genotype; * interaction effect genotype x time. Significant results from uncorrected two-sample tests are depicted by a red bar on the x-axis.

(A) Graphical representation of phase-based connectivity. Phase-based connectivity refers to the correlation of relative phase between two oscillatory signals. In the current study, the wPLI was analyzed for frontal cortex – parietal cortex connections (dark blue) and for frontal cortex – hippocampus connections (purple).

(B) Frontal cortex – parietal cortex wPLI spectrogram for 3-month-old (left) and 12-month-old (right) mice.

(C) Frontal cortex – hippocampus wPLI spectrogram for 3-month-old (left) and 12-month-old (right) mice.

(D) Frontal cortex – parietal cortex average wPLI per frequency band over the 9-month recording period. Frequency bands from left to right: delta (1-5 Hz), theta/alpha (5-13 Hz), beta 1 (13-20 Hz), beta 2 (20-30 Hz), gamma (30-50 Hz). θ/α: ^

(E) Frontal cortex – hippocampus average wPLI per frequency band over the 9-month recording period. Frequency bands from left to right: delta (1-5 Hz), theta/alpha (5-13 Hz), beta 1 (13-20 Hz), beta 2 (20-30 Hz), gamma (30-50 Hz). No main effect of genotype or genotype x time interaction effect was found.

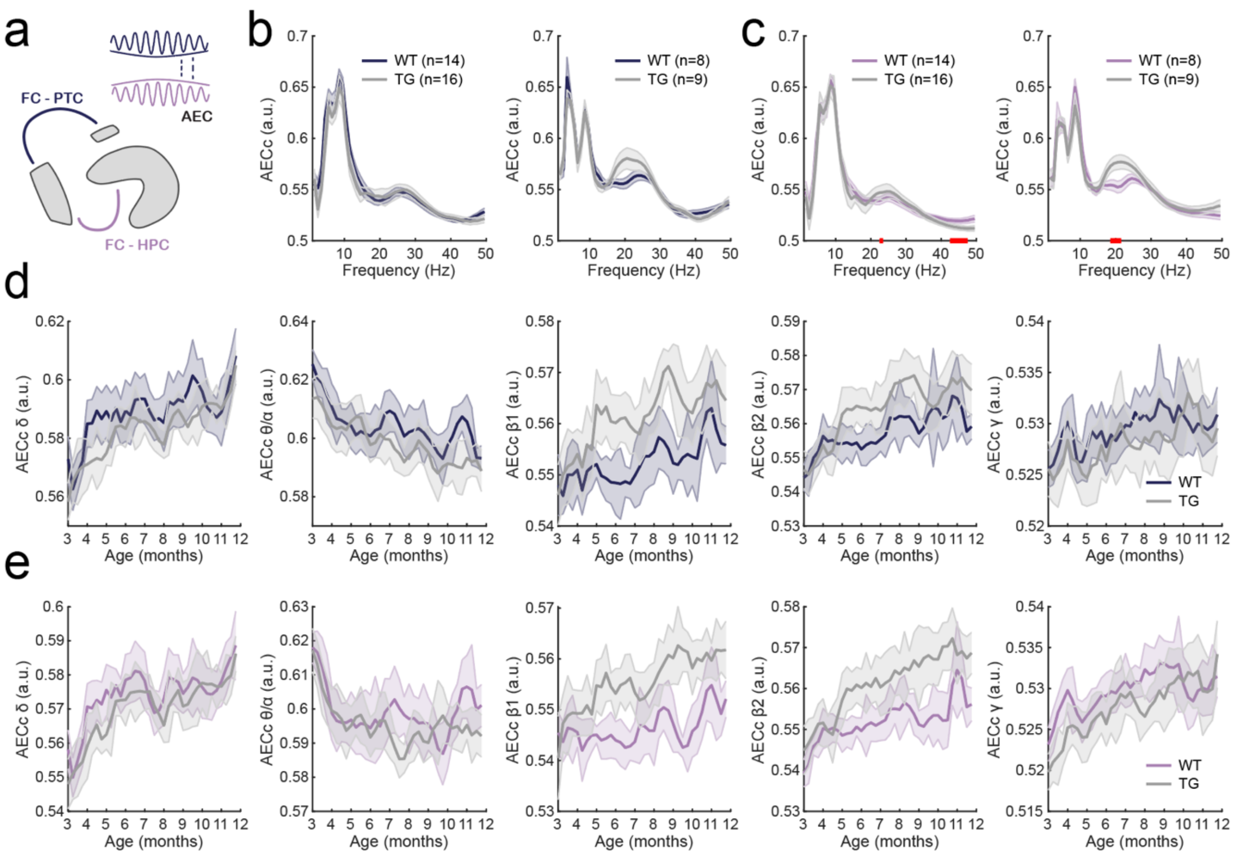

Supplementary Figure 10. AD-related changes in amplitude-based connectivity, as measured by amplitude envelope correlation (AEC), in APP/PS1 mice during quiet wake. Results of mixed-effects analysis are referred to in legends: ^ main effect of genotype; * interaction effect genotype x time. Significant results from uncorrected two-sample tests are depicted by a red bar on the x-axis.

(A) Graphical representation of amplitude-based connectivity. Phase-based connectivity refers to the correlation of the amplitude of two oscillatory signals. In the current study, the corrected AEC (AECc) was analyzed for frontal cortex (FC) – parietal cortex connections (PTC) (dark blue) and for frontal cortex – hippocampus (HPC) connections (purple).

(B) Frontal cortex – parietal cortex AECc spectrogram for 3-month-old (left) and 12-month-old (right) mice.

(C) Frontal cortex – hippocampus AECc spectrogram for 3-month-old (left) and 12-month-old (right) mice.

(D) Frontal cortex – parietal cortex average AECc per frequency band over the 9-month recording period. Frequency bands from left to right: delta (1-5 Hz), theta/alpha (5-13 Hz), beta 1 (13-20 Hz), beta 2 (20-30 Hz), gamma (30-50 Hz).

(E) Frontal cortex – hippocampus average AECc per frequency band over the 9-month recording period. Frequency bands from left to right: delta (1-5 Hz), theta/alpha (5-13 Hz), beta 1 (13-20 Hz), beta 2 (20-30 Hz), gamma (30-50 Hz). β1: ^, β2: ^

SUPPLEMENTARY TABLES

Supplementary Table 1. ***APP*** **and** ***PSEN1*** **mutations of pre-symptomatic human mutation carriers.**

Gene	Nucleotide change	Protein change
APP	duplication	
PSEN1	c.236 C>T	p.(Ala79Val)
PSEN1	c.344 A>G	p.(Tyr115Cys)
PSEN1	c.786 G>C	p.(Leu262Phe)
PSEN1	c.692 C>T	p.(Ala231Val)
PSEN1	c.791 C>T	p.(Pro264Leu)

Supplementary Table 2. Subdivision of regions of interest (ROIs) as in the automatic anatomical labeling atlas (AAL).

ROI #	Name
Frontal (L)	
1	Rectus Gyrus
2	Olfactory Gyrus
3	Sup. Frontal Orbital
4	Med. Frontal Orbital
5	Mid. Frontal Orbital
6	Inf. Frontal Orbital
7	Sup. Frontal Gyrus
8	Mid. Frontal Gyrus
9	Inf. Frontal Opercular
10	Inf. Frontal Triangular
11	Med. Sup. Frontal Gyrus
Parietal (L)	
17	Sup. Parietal Gyrus
18	Inf. Parietal Gyrus
19	Supramarginal Gyrus
20	Angular Gyrus
21	Precuneus
Hippocampus (L)	
79	Hippocampus

Supplementary Table 3. Mixed-effects analysis of velocity. QW: Quiet wake; AM: Awake moving; PFC: prefrontal cortex; PTC: parietal cortex; HPC: hippocampus.

**Behavioral state**	**Type**	**Time**	**P value**	**Genotype**	**P value**	**Time x Genotype**	**P value**
All	Velocity	F (6,75, 130) = 4,17	<,001	F (1, 28) = 0,779	0,385	F (35, 675) = 1,24	0,168
QW	Percentage	F (8,56, 165) = 3,88	<,001	F (1, 28) = 0,998	0,326	F (35, 673) = 1,36	0,085
AM	Percentage	F (10,3, 199) = 2,09	0,025	F (1, 28) = 0,205	0,654	F (35, 672) = 0,920	0,602
QW	Velocity	F (7,50, 145) = 4,28	<,001	F (1, 28) = 0,678	0,417	F (35, 675) = 1,20	0,197
AM	Velocity	F (9,61, 185) = 3,38	<,001	F (1, 28) = 0,149	0,702	F (35, 675) = 0,937	0,575

Supplementary Table 4. Neuropsychological, subjective cognitive decline and psychiatric test scores. Each column presents test scores of one *APP* or *PSEN1* mutation carrier. Global cognition is presented by total MMSE score. When applicable, neuropsychological test scores were converted to *t*-scores (RAVL, Rey CF recall, Digit span, TMT, Stroop, Fluency) or percentiles (VAT, Rey CF copy,). Raw test scores were shown for VAT naming (max 12) and VOSP number location (max 10). Tests are ordered according to cognitive domain (memory, attention, executive functioning, language, visuospatial functioning). Raw test scores were also shown for additional questionnaires SCF, CCI and HADS. IADL *t*-scores were obtained using the Item Response Theory method. Abnormal test scores are indicated in grey. None of the mutation carriers showed clear abnormal functioning on any of the cognitive domains. One mutation carrier had mild attention problems, which could be explained by the level of anxiety. MMSE: Mini-Mental State Examination; VAT: Visual Association Test; RAVL: Dutch version of Rey Auditory Verbal Learning Test; R/T: *t*-score Recall corrected for Total score; Rey CF: Rey Complex Figure Task; TMT: Trail Making Test; Stroop: Stroop color word test; Fluency: Letter (version D-A-T) and category animal fluency test; VOSP: Visual Object and Space Perception Battery (Nr loc: number location); SCF: Subjective Cognitive Functioning questionnaire; CCI: Cognitvie Change Index; HADS: Hospital Anxiety and Depression Scale; A: Anxiety; D: Depression; IADL: Amsterdam questionnaire of instrumental activities of daily living in (early) dementia; *na*: not available.

**Global**	MMSE		30	30	29	28	30	28	28	27	27	29	30
**Memory**	VAT	Total	20	20	20	20	20	20	20	20	20	20	20
	RAVL	Total	53	58	53	70	56	34	43	70	45	35	41
		Recall	49	62	58	68	62	40	47	64	40	38	46
		R/T	42	57	57	51	60	52	53	45	40	52	56
	Rey CF	Recall	*na*	49	42	*na*	73	65	43	40	60	36	34
**Attention**	Digit span	Forward	*na*	50	61	*na*	54	72	50	27	74	54	58
	TMT	A	39	64	49	40	57	52	45	36	55	64	54
	Stroop	1	36	64	30	57	59	31	33	36	57	46	41
		2	37	55	29	72	42	32	38	30	52	37	52
**Executive functioning**	TMT	B	39	67	44	45	50	50	39	31	36	49	67
	Digit span	Backward	*na*	70	48	*na*	64	61	52	48	67	50	68
	Stroop	3	40	59	36	38	45	53	31	41	58	43	58
	Fluency	DAT	42	62	55	57	55	60	66	55	63	46	25
**Language**	Fluency	Animal	40	58	46	65	65	50	52	42	61	58	39
	VAT	Naming	12	12	12	12	12	12	12	12	12	12	12
**Visuospatial functioning**	Rey CF	Copy	*na*	>16	<1	*na*	>16	>16	>16	>16	>16	>16	>16
	VOSP	Nr location	*na*	7	7	*na*	10	10	10	9	*na*	9	10
**Subjective decline**	SCF	Self	0	-1	-3	0	0	-2	-1	-3	0	0	-3
	CCI	Self	23	32	34	27	20	20	20	45	20	20	22
	CCI	Informant	40	23	23	28	20	21	20	39	20	20	25
**Psychiatric symptoms**	HADS	A	9	10	5	1	9	4	6	15	5	4	12
		D	*na*	5	4	5	0	2	2	7	0	0	9
**Interference**	iADL	Informant	63	70	55	68	69	69	69	53	69	70	68

Supplementary Table 5. Mixed-effects analysis of absolute power. QW: Quiet wake; AM: Awake moving; PFC: prefrontal cortex; PTC: parietal cortex; HPC: hippocampus.

Behavioral state	Brain region	Frequency range	Time	P value	Genotype	P value	Time x Genotype	P value
QW	PFC	1-120 Hz	F (3,62, 69,8) = 8,55	<,001	F (1, 28) = 0,103	0,750	F (35, 675) = 0,817	0,766
QW	PTC	1-120 Hz	F (3,06, 58,9) = 8,46	<,001	F (1, 28) = 1,13	0,298	F (35, 675) = 1,55	0,024
QW	HPC	1-120 Hz	F (2,56, 49,3) = 7,69	<,001	F (1, 28) = 0,0158	0,901	F (35, 675) = 3,08	<,001
AM	PFC	1-120 Hz	F (2,79, 53,9) = 6,54	<,001	F (1, 28) = 0,0546	0,817	F (35, 676) = 0,694	0,909
AM	PTC	1-120 Hz	F (2,58, 49,9) = 9,58	<,001	F (1, 28) = 0,867	0,360	F (35, 676) = 1,84	0,003
AM	HPC	1-120 Hz	F (2,31, 44,5) = 8,41	<,001	F (1, 28) = 0,00555	0,941	F (35, 676) = 3,17	<,001
QW	PFC	δ	F (5,22, 101) = 9,62	<,001	F (1, 28) = 0,00504	0,944	F (35, 675) = 1,08	0,349
QW	PFC	θ	F (4,67, 90,0) = 13,5	<,001	F (1, 28) = 0,546	0,466	F (35, 675) = 0,824	0,755
QW	PFC	α	F (4,42, 85,2) = 1,71	0,149	F (1, 28) = 0,473	0,497	F (35, 675) = 0,836	0,738
QW	PFC	β	F (3,02, 58,2) = 3,60	0,018	F (1, 28) = 13,1	0,001	F (35, 675) = 1,97	<,001
QW	PFC	γ1	F (2,35, 45,4) = 4,34	0,014	F (1, 28) = 3,48	0,073	F (35, 675) = 1,51	0,032
QW	PFC	√2	F (7,33, 141) = 5,43	<,001	F (1, 28) = 2,40	0,133	F (35, 675) = 0,790	0,803
QW	PTC	δ	F (5,23, 101) = 8,64	<,001	F (1, 28) = 2,60	0,118	F (35, 675) = 1,35	0,086
QW	PTC	θ	F (3,17, 61,2) = 5,68	0,001	F (1, 28) = 0,184	0,671	F (35, 675) = 1,14	0,268
QW	PTC	α	F (4,03, 77,8) = 11,8	<,001	F (1, 28) = 0,773	0,387	F (35, 675) = 2,08	<,001
QW	PTC	β	F (3,74, 72,2) = 12,8	<,001	F (1, 28) = 1,89	0,180	F (35, 675) = 3,51	<,001
QW	PTC	γ1	F (3,77, 72,7) = 19,0	<,001	F (1, 28) = 2,27	0,143	F (35, 675) = 3,16	<,001
QW	PTC	√2	F (4,51, 86,9) = 15,1	<,001	F (1, 28) = 0,173	0,681	F (35, 675) = 0,979	0,504
QW	HPC	δ	F (4,88, 94,1) = 3,31	0,009	F (1, 28) = 1,10	0,303	F (35, 675) = 1,21	0,194
QW	HPC	θ	F (3,76, 72,6) = 4,16	0,005	F (1, 28) = 0,00298	0,957	F (35, 675) = 2,14	<,001
QW	HPC	α	F (3,57, 68,9) = 10,1	<,001	F (1, 28) = 0,213	0,648	F (35, 675) = 3,34	<,001
QW	HPC	β	F (2,81, 54,3) = 9,87	<,001	F (1, 28) = 0,562	0,460	F (35, 675) = 3,81	<,001
QW	HPC	γ1	F (2,08, 40,0) = 18,6	<,001	F (1, 28) = 0,0160	0,900	F (35, 675) = 4,22	<,001
QW	HPC	√2	F (2,33, 45,0) = 12,3	<,001	F (1, 28) = 1,80	0,191	F (35, 675) = 1,92	0,001
AM	PFC	δ	F (3,30, 63,7) = 7,88	<,001	F (1, 28) = 0,197	0,660	F (35, 676) = 0,665	0,932
AM	PFC	θ	F (4,42, 85,4) = 12,7	<,001	F (1, 28) = 1,30	0,265	F (35, 676) = 0,946	0,560
AM	PFC	α	F (3,74, 72,3) = 2,58	0,048	F (1, 28) = 0,146	0,706	F (35, 676) = 0,776	0,821
AM	PFC	β	F (3,12, 60,4) = 2,17	0,099	F (1, 28) = 15,5	<,001	F (35, 676) = 1,65	0,012
AM	PFC	γ1	F (2,11, 40,7) = 2,61	0,083	F (1, 28) = 5,19	0,031	F (35, 676) = 1,26	0,148
AM	PFC	√2	F (5,75, 111) = 4,70	<,001	F (1, 28) = 3,87	0,059	F (35, 676) = 0,661	0,935
AM	PTC	δ	F (4,40, 84,9) = 9,42	<,001	F (1, 28) = 1,55	0,224	F (35, 676) = 0,933	0,582
AM	PTC	θ	F (2,60, 50,2) = 6,54	0,001	F (1, 28) = 0,161	0,692	F (35, 676) = 1,68	0,009
AM	PTC	α	F (3,55, 68,5) = 11,4	<,001	F (1, 28) = 0,397	0,534	F (35, 676) = 1,63	0,014
AM	PTC	β	F (3,21, 62,0) = 8,99	<,001	F (1, 28) = 2,10	0,159	F (35, 676) = 2,58	<,001
AM	PTC	γ1	F (3,28, 63,4) = 17,2	<,001	F (1, 28) = 3,20	0,085	F (35, 676) = 2,83	<,001
AM	PTC	√2	F (3,43, 66,3) = 15,4	<,001	F (1, 28) = 0,255	0,617	F (35, 676) = 0,971	0,519
AM	HPC	δ	F (3,73, 72,1) = 3,86	0,008	F (1, 28) = 0,191	0,666	F (35, 676) = 1,62	0,015
AM	HPC	θ	F (3,10, 59,8) = 4,63	0,005	F (1, 28) = 0,0117	0,915	F (35, 676) = 3,00	<,001
AM	HPC	α	F (3,20, 61,8) = 10,3	<,001	F (1, 28) = 0,00772	0,931	F (35, 676) = 2,66	<,001
AM	HPC	β	F (2,70, 52,1) = 7,07	<,001	F (1, 28) = 1,15	0,293	F (35, 676) = 2,39	<,001
AM	HPC	γ1	F (2,13, 41,1) = 15,2	<,001	F (1, 28) = 0,0383	0,846	F (35, 676) = 3,61	<,001
AM	HPC	√2	F (2,08, 40,1) = 12,1	<,001	F (1, 28) = 1,20	0,283	F (35, 676) = 2,11	<,001

Supplementary Table 6. Mixed-effects analysis of relative power. QW: Quiet wake; AM: Awake moving; PFC: prefrontal cortex; PTC: parietal cortex; HPC: hippocampus.

**Behavioral state**	**Brain region**	**Frequency range**	**Time**	**P value**	**Genotype**	**P value**	**Time x Genotype**	**P value**
QW	PFC	δ	F (6,54, 126) = 5,70	<,001	F (1, 28) = 0,729	0,401	F (35, 675) = 1,51	0,032
QW	PFC	θ	F (8,70, 168) = 10,7	<,001	F (1, 28) = 6,03	0,021	F (35, 675) = 0,932	0,583
QW	PFC	α	F (7,10, 137) = 8,00	<,001	F (1, 28) = 0,502	0,484	F (35, 675) = 0,705	0,899
QW	PFC	β	F (7,92, 153) = 13,7	<,001	F (1, 28) = 8,24	0,008	F (35, 675) = 1,49	0,035
QW	PFC	γ1	F (5,95, 115) = 23,2	<,001	F (1, 28) = 1,58	0,219	F (35, 675) = 1,98	<,001
QW	PFC	√2	F (4,26, 82,1) = 26,6	<,001	F (1, 28) = 1,75	0,197	F (35, 675) = 0,983	0,498
QW	PTC	δ	F (8,61, 166) = 13,4	<,001	F (1, 28) = 0,518	0,478	F (35, 675) = 1,31	0,112
QW	PTC	θ	F (6,32, 122) = 1,48	0,187	F (1, 28) = 5,59	0,025	F (35, 675) = 0,985	0,496
QW	PTC	α	F (9,84, 190) = 11,9	<,001	F (1, 28) = 0,117	0,735	F (35, 675) = 0,992	0,484
QW	PTC	β	F (7,77, 150) = 11,9	<,001	F (1, 28) = 5,47	0,027	F (35, 675) = 1,98	<,001
QW	PTC	γ1	F (8,09, 156) = 20,4	<,001	F (1, 28) = 5,81	0,023	F (35, 675) = 1,05	0,395
QW	PTC	√2	F (3,83, 73,9) = 9,99	<,001	F (1, 28) = 0,703	0,409	F (35, 675) = 2,00	<,001
QW	HPC	δ	F (5,30, 102) = 14,6	<,001	F (1, 28) = 1,09	0,305	F (35, 675) = 1,28	0,129
QW	HPC	θ	F (5,56, 107) = 5,23	<,001	F (1, 28) = 0,238	0,630	F (35, 675) = 0,754	0,848
QW	HPC	α	F (8,76, 169) = 7,23	<,001	F (1, 28) = 1,34	0,257	F (35, 675) = 1,17	0,233
QW	HPC	β	F (5,29, 102) = 7,25	<,001	F (1, 28) = 6,39	0,017	F (35, 675) = 1,95	0,001
QW	HPC	γ1	F (3,79, 73,1) = 26,5	<,001	F (1, 28) = 0,202	0,657	F (35, 675) = 1,75	0,005
QW	HPC	√2	F (3,40, 65,7) = 9,30	<,001	F (1, 28) = 6,96	0,013	F (35, 675) = 0,637	0,950
AM	PFC	δ	F (3,20, 61,8) = 4,80	0,004	F (1, 28) = 3,65	0,066	F (35, 676) = 0,896	0,643
AM	PFC	θ	F (5,49, 106) = 14,5	<,001	F (1, 28) = 5,44	0,027	F (35, 676) = 0,925	0,595
AM	PFC	α	F (6,81, 131) = 7,60	<,001	F (1, 28) = 0,317	0,578	F (35, 676) = 0,633	0,952
AM	PFC	β	F (7,31, 141) = 10,2	<,001	F (1, 28) = 15,6	<,001	F (35, 676) = 1,40	0,064
AM	PFC	γ1	F (5,73, 111) = 26,4	<,001	F (1, 28) = 4,43	0,044	F (35, 676) = 1,19	0,209
AM	PFC	√2	F (3,73, 72,1) = 29,8	<,001	F (1, 28) = 3,38	0,076	F (35, 676) = 0,773	0,825
AM	PTC	δ	F (5,40, 104) = 12,4	<,001	F (1, 28) = 0,0153	0,903	F (35, 676) = 1,17	0,229
AM	PTC	θ	F (4,19, 81,0) = 1,91	0,114	F (1, 28) = 4,97	0,034	F (35, 676) = 1,17	0,229
AM	PTC	α	F (9,25, 179) = 7,45	<,001	F (1, 28) = 0,335	0,567	F (35, 676) = 0,883	0,664
AM	PTC	β	F (7,37, 142) = 4,39	<,001	F (1, 28) = 8,11	0,008	F (35, 676) = 1,52	0,028
AM	PTC	γ1	F (5,99, 116) = 12,8	<,001	F (1, 28) = 18,3	<,001	F (35, 676) = 0,863	0,696
AM	PTC	√2	F (2,43, 47,0) = 5,21	0,006	F (1, 28) = 0,346	0,561	F (35, 676) = 2,32	<,001
AM	HPC	δ	F (3,30, 63,7) = 10,5	<,001	F (1, 28) = 0,257	0,616	F (35, 676) = 1,48	0,038
AM	HPC	θ	F (4,51, 87,0) = 6,96	<,001	F (1, 28) = 1,06	0,312	F (35, 676) = 0,879	0,670
AM	HPC	α	F (7,64, 148) = 5,11	<,001	F (1, 28) = 0,0116	0,915	F (35, 676) = 1,00	0,467
AM	HPC	β	F (5,82, 112) = 4,10	0,001	F (1, 28) = 14,0	<,001	F (35, 676) = 1,06	0,383
AM	HPC	γ1	F (3,55, 68,5) = 17,5	<,001	F (1, 28) = 0,578	0,454	F (35, 676) = 1,21	0,192
AM	HPC	√2	F (2,90, 56,1) = 7,01	<,001	F (1, 28) = 4,96	0,034	F (35, 676) = 0,322	>,999

Supplementary Table 7. Mixed-effects analysis of theta peak frequency. QW: Quiet wake; AM: Awake moving; PFC: prefrontal cortex; PTC: parietal cortex; HPC: hippocampus.

**Behavioral state**	**Brain region**	**Time**	**P value**	**Genotype**	**P value**	**Time x Genotype**	**P value**
QW	PTC	F (10,3, 187) = 2,79	0,003	F (1, 28) = 0,248	0,622	F (35, 635) = 0,971	0,517
QW	HPC	F (9,32, 176) = 3,00	0,002	F (1, 28) = 0,185	0,670	F (35, 661) = 1,08	0,348
AM	PTC	F (8,36, 161) = 3,87	<,001	F (1, 28) = 2,28	0,142	F (35, 676) = 1,04	0,406
AM	HPC	F (8,93, 172) = 3,99	<,001	F (1, 28) = 2,24	0,146	F (35, 676) = 0,949	0,555

Supplementary Table 8. Mixed-effects analysis of wPLI. QW: Quiet wake; AM: Awake moving; PFC: prefrontal cortex; PTC: parietal cortex; HPC: hippocampus.

Behavioral state	Connection	Frequency range	Time	P value	Genotype	P value	Time x Genotype	P value
QW	PFC-PTC	δ	F (8,59, 165) = 0,947	0,483	F (1, 28) = 2,25	0,145	F (35, 673) = 0,561	0,982
QW	PFC-PTC	θ/a	F (9,07, 174) = 5,28	<,001	F (1, 28) = 9,68	0,004	F (35, 673) = 1,26	0,151
QW	PFC-PTC	β1	F (6,66, 128) = 0,932	0,481	F (1, 28) = 1,53	0,227	F (35, 673) = 0,824	0,755
QW	PFC-PTC	β2	F (5,72, 110) = 0,820	0,552	F (1, 28) = 0,471	0,498	F (35, 673) = 0,800	0,790
QW	PFC-PTC	γ	F (7,08, 136) = 1,01	0,426	F (1, 28) = 1,81	0,189	F (35, 673) = 0,735	0,869
QW	PFC-HPC	δ	F (9,17, 176) = 1,36	0,209	F (1, 28) = 0,277	0,603	F (35, 673) = 0,771	0,827
QW	PFC-HPC	θ/a	F (7,39, 142) = 4,43	<,001	F (1, 28) = 2,46	0,128	F (35, 673) = 0,956	0,544
QW	PFC-HPC	β1	F (7,48, 144) = 1,17	0,322	F (1, 28) = 0,155	0,697	F (35, 673) = 1,40	0,065
QW	PFC-HPC	β2	F (8,45, 162) = 1,05	0,403	F (1, 28) = 0,215	0,647	F (35, 673) = 1,04	0,405
QW	PFC-HPC	γ	F (5,43, 104) = 1,02	0,414	F (1, 28) = 2,07	0,161	F (35, 673) = 1,18	0,226
AM	PFC-PTC	δ	F (8,29, 160) = 1,04	0,411	F (1, 28) = 0,718	0,404	F (35, 674) = 0,884	0,663
AM	PFC-PTC	θ/a	F (9,04, 174) = 5,62	<,001	F (1, 28) = 14,1	<,001	F (35, 674) = 1,66	0,011
AM	PFC-PTC	β1	F (5,96, 115) = 0,992	0,434	F (1, 28) = 1,55	0,223	F (35, 674) = 0,762	0,838
AM	PFC-PTC	β2	F (5,75, 111) = 1,50	0,186	F (1, 28) = 0,0502	0,824	F (35, 674) = 0,716	0,889
AM	PFC-PTC	γ	F (4,50, 86,7) = 0,837	0,517	F (1, 28) = 1,47	0,236	F (35, 674) = 0,760	0,841
AM	PFC-HPC	δ	F (8,82, 170) = 1,17	0,317	F (1, 28) = 0,619	0,438	F (35, 674) = 0,959	0,539
AM	PFC-HPC	θ/a	F (7,80, 150) = 3,87	<,001	F (1, 28) = 4,22	0,049	F (35, 674) = 1,11	0,306
AM	PFC-HPC	β1	F (7,24, 139) = 0,957	0,467	F (1, 28) = 0,000274	0,987	F (35, 674) = 1,08	0,351
AM	PFC-HPC	β2	F (8,04, 155) = 1,25	0,272	F (1, 28) = 0,0987	0,756	F (35, 674) = 0,797	0,794
AM	PFC-HPC	γ	F (4,42, 85,1) = 1,32	0,267	F (1, 28) = 3,32	0,079	F (35, 674) = 1,29	0,124

Supplementary Table 9. Mixed-effects analysis of AECc. QW: Quiet wake; AM: Awake moving; PFC: prefrontal cortex; PTC: parietal cortex; HPC: hippocampus.

Behavioral state	Connection	Frequency range	Time	P value	Genotype	P value	Time x Genotype	P value
QW	PFC-PTC	δ	F (8,65, 153) = 3,97	<,001	F (1, 28) = 2,31	0,14	F (35, 619) = 0,886	0,659
QW	PFC-PTC	θ/a	F (7,52, 133) = 4,45	<,001	F (1, 28) = 0,0516	0,822	F (35, 619) = 1,05	0,388
QW	PFC-PTC	β1	F (7,12, 126) = 3,06	0,005	F (1, 28) = 3,49	0,072	F (35, 619) = 0,861	0,699
QW	PFC-PTC	β2	F (7,79, 138) = 4,74	<,001	F (1, 28) = 1,31	0,263	F (35, 619) = 0,755	0,847
QW	PFC-PTC	γ	F (7,86, 139) = 2,54	0,013	F (1, 28) = 0,0362	0,85	F (35, 619) = 0,862	0,698
QW	PFC-HPC	δ	F (9,42, 167) = 4,15	<,001	F (1, 28) = 1,70	0,203	F (35, 619) = 0,646	0,944
QW	PFC-HPC	θ/a	F (7,04, 125) = 3,31	0,003	F (1, 28) = 0,00490	0,945	F (35, 619) = 0,954	0,546
QW	PFC-HPC	β1	F (5,63, 99,5) = 1,82	0,107	F (1, 28) = 6,74	0,015	F (35, 619) = 1,12	0,294
QW	PFC-HPC	β2	F (7,27, 129) = 4,55	<,001	F (1, 28) = 6,71	0,015	F (35, 619) = 1,13	0,28
QW	PFC-HPC	γ	F (7,21, 128) = 3,36	0,002	F (1, 28) = 0,0484	0,827	F (35, 619) = 1,32	0,105
AM	PFC-PTC	δ	F (7,94, 132) = 8,21	<,001	F (1, 28) = 0,882	0,356	F (35, 581) = 0,822	0,758
AM	PFC-PTC	θ/a	F (5,28, 87,7) = 8,84	<,001	F (1, 28) = 0,259	0,615	F (35, 581) = 1,27	0,139
AM	PFC-PTC	β1	F (5,69, 94,5) = 2,71	0,02	F (1, 28) = 5,24	0,03	F (35, 581) = 0,745	0,858
AM	PFC-PTC	β2	F (5,19, 86,1) = 6,25	<,001	F (1, 28) = 2,47	0,127	F (35, 581) = 1,26	0,15
AM	PFC-PTC	γ	F (4,12, 68,4) = 1,75	0,147	F (1, 28) = 2,46	0,128	F (35, 581) = 1,00	0,463
AM	PFC-HPC	δ	F (7,55, 125) = 9,12	<,001	F (1, 28) = 0,783	0,384	F (35, 581) = 0,726	0,878
AM	PFC-HPC	θ/a	F (4,14, 68,7) = 3,76	0,007	F (1, 28) = 0,0308	0,862	F (35, 581) = 1,05	0,395
AM	PFC-HPC	β1	F (4,82, 79,9) = 1,94	0,099	F (1, 28) = 7,27	0,012	F (35, 581) = 0,800	0,789
AM	PFC-HPC	β2	F (4,15, 69,0) = 3,00	0,023	F (1, 28) = 6,92	0,014	F (35, 581) = 1,68	0,009
AM	PFC-HPC	γ	F (4,61, 76,6) = 1,98	0,096	F (1, 28) = 2,76	0,108	F (35, 581) = 0,592	0,971
